# Comparative transcriptomic analysis of two *Cucumis melo* var. *saccharinu*s germplasms differing in fruit physical and chemical characteristics

**DOI:** 10.1186/s12870-022-03550-8

**Published:** 2022-04-12

**Authors:** Renfan Liang, Yicheng Su, Xiaojuan Qin, Zhongkui Gao, Zhixin Fu, Huijun Qiu, Xu Lin, Jinlian Zhu

**Affiliations:** 1 Guangxi Academy of Agricultural Sciences, Nanning, 530007 China; 2 Guangxi Normal University for Nationalities, Chongzuo, 532200 China

**Keywords:** *Cucumis melo* var, *Saccharinu*s, Soluble sugar, Physical characteristics, Fruit ripening, Transcriptome

## Abstract

**Background:**

Hami melon (*Cucumis melo* var. *saccharinus*) is a popular fruit in China because of its excellent taste, which is largely determined by its physicochemical characteristics, including flesh texture, sugar content, aroma, and nutrient composition. However, the mechanisms by which these characteristics are regulated have not yet been determined. In this study, we monitored changes in the fruits of two germplasms that differed in physicochemical characteristics throughout the fruit development period.

**Results:**

Ripe fruit of the bred variety ‘Guimi’ had significantly higher soluble sugar contents than the fruit of the common variety ‘Yaolong.’ Additionally, differences in fruit shape and color between these two germplasms were observed during development. Comparative transcriptome analysis, conducted to identify regulators and pathways underlying the observed differences at corresponding stages of development, revealed a higher number of differentially expressed genes (DEGs) in Guimi than in Yaolong. Moreover, most DEGs detected during early fruit development in Guimi were associated with cell wall biogenesis. Temporal analysis of the identified DEGs revealed similar trends in the enrichment of downregulated genes in both germplasms, although there were differences in the enrichment trends of upregulated genes. Further analyses revealed trends in differential changes in multiple genes involved in cell wall biogenesis and sugar metabolism during fruit ripening.

**Conclusions:**

We identified several genes associated with the ripening of Hami melons, which will provide novel insights into the molecular mechanisms underlying the development of fruit characteristics in these melons.

**Supplementary Information:**

The online version contains supplementary material available at 10.1186/s12870-022-03550-8.

## Background

The Hami melon (*Cucumis melo* var. *saccharinus*) is among the most important diploid crops within the family Cucurbitaceae and its different cultivars are characterized by highly variable fruit traits such as flesh color, sugar content, and shape [1]. The production of Hami melons in Guangxi Province, China, began in the middle to late 1990s [2]. However, given the small differences in day and night temperatures, the Guangxi environment is not particularly conducive to sugar accumulation in Hami melon, as it is difficult to achieve the desired 14% soluble solid content [3]. In addition, high temperatures and humidity often contribute to the development of a range of diseases and pest infestations, thereby reducing yields and resulting in heavy economic losses, notably as a consequence of gummy stem blight caused by *Mycosphaerella melonis*. To address these problems, ‘Guimi-12,’ a new high-quality Guangxi germplasm characterized by high yields and disease resistance, has been selected and popularized in the region. Guimi-12 differs from the common Hami melon germplasm (‘Yaolong’) in shape, size, flesh texture, sugar content, aroma, and nutrient composition, which are important factors in the breeding of fruit-bearing plants and are the focus of studies to identify and characterize the associated regulatory transcription factors and molecular mechanisms [4]. However, little is known about the differences between these two Hami melon varieties with respect to gene expression patterns and regulatory pathways, especially those related to sugar metabolism.

To date, breeding programs for Hami melon have focused primarily on the selection of traits associated with fruit thickness and sweetness [5]. Pericarp hardening is generally associated with the development of secondary cell wall structures, although the underlying mechanisms have yet to be ascertained. However, it has been established that a transcription network comprising NAC and MYB transcription factors is highly conserved in the regulation of pericarp formation and secondary cell wall development in *Arabidopsis* [6]. Regarding sweetness, sucrose was identified as the predominant sugar in ripe melon fruit [7, 8], and α-galactosidase (α-Gal) as the initial enzyme in the decomposition of stachyose. α-Gal activity was shown to be closely associated with the accumulation of fructose and glucose during fruit expansion [9]. Moreover, acidic α-Gal has been found to have a higher activity in the sweet tissue of watermelon fruit than in the unsweet tissue, thereby highlighting its significance in the accumulation of sugars in watermelon [10]. Numerous metabolic pathways contribute to sugar accumulation in watermelon fruit [11], with 62 sugar synthesis enzymes and 76 sugar transporter genes identified as being associated with these pathways [4], of which 13 and 14 genes, respectively, were differentially expressed during fruit development [12]. In addition, the patterns of sugar accumulation during fruit maturation differed between oriental and other types of melon [13]. These observations indicate that differences in the genes and pathways associated with sugar metabolism probably determine the differing sucrose content of the fruits. Thus, we speculate that sugar content in the fruit of Guimi-12 Hami melons might also differ from those in Yaolong Hami melons.

Transcriptomes are collections of all transcripts produced by a species or specific types of organs, tissues, and cells, and include the number of transcripts, expressional dynamics at specific developmental stages, post-transcriptional modification, and regulation of non-coding RNAs [14]. By conducting transcriptomic studies, more comprehensive analyses of gene expression, structure, and function in specific species can be performed, thereby providing a complete understanding of the molecular mechanisms underlying specific physiological characteristics [15]. Compared with the whole genome, properties at the transcriptome level tend to be temporally and spatially discrete [16, 17]. Thus, transcriptome sequencing technology can provide a new approach for studying gene function and physiological characteristics in Hami melons. In this study, we comparatively analyzed the transcriptomes of two contrasting Hami melon germplasms, namely, Guimi-12 and Yaolong, throughout the fruit ripening process, and identified genes that play roles in the development and ripening of Hami melon fruit. These findings provide further insights into the identification of key pathways and regulators associated with the physicochemical characteristics of Hami melon fruit.

## Results

### Variations in the physicochemical characteristics of melon fruit during ripening

**Fig. 1:**
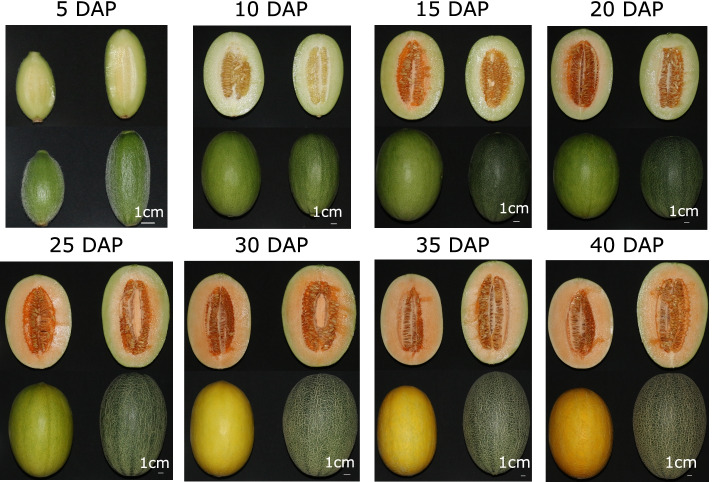
Fruits of the Guimi and Yaolong varieties of Hami melon at different stages of development. For each sampling time image, Guimi and Yaolong fruits are depicted on the left- and right-hand sides, respectively

To compare the fruits of the two assessed Hami melon varieties, we examined the physicochemical characteristics of the fruits collected at different stages of development (Fig. 1). The color of the fruit peel of the two varieties was similar at 5 and 10 days after pollination (DAP). However, the peel of the Yaolong fruit turned darker green than that of Guimi from 15 to 20 DAP. With continued growth, Guimi melons developed into medium-sized oval fruits with yellow skin. In both varieties, juicy flesh underwent a change in color from white to yellow during ripening.

**Fig. 2:**
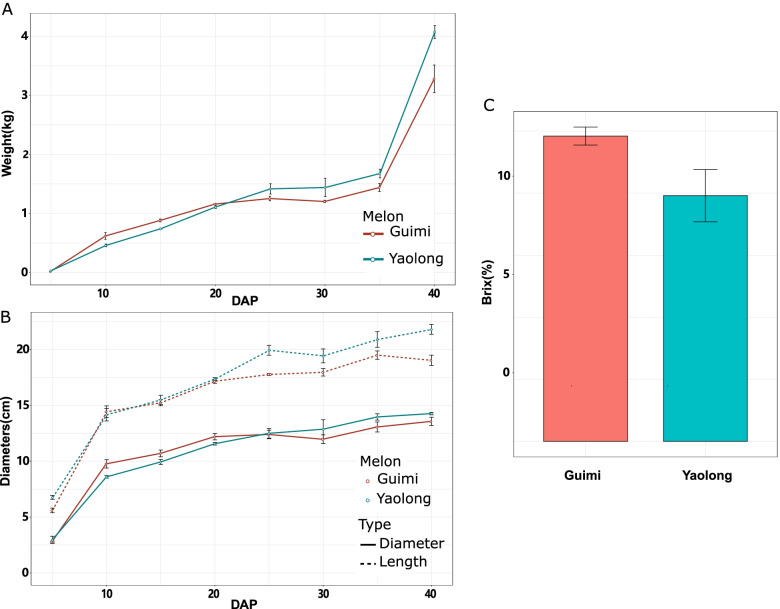
Differences in the weight, size, and soluble sugar contents of fruits of the Guimi and Yaolong varieties of Hami melon. Trends in weight (**A**) and size (length, diameter) (**B**) at 5, 10, 15, 20, 25, 30, 35, and 40 days after pollination (DAP). (**C**) Total soluble sugar contents at 40 DAP. Data represent the means of three individual replicates. The bars denote standard error values (*n* = 3)

Trends in changes in the physicochemical characteristics of the fruits, including weight, size, and soluble sugar content, are shown in Fig. 2. Similar trends were observed in the two varieties with respect to the weight and size of the fruit during ripening. Interestingly, fruit weight increased rapidly from 35 to 40 DAP, whereas there was only a slight increase in fruit size. At 40 DAP, the soluble sugar content of Guimi fruit reached a value of 12, which was higher than that obtained for Yaolong, although differences in the Brix values of the two varieties were non-significant.

### Global analysis of the RNA-Seq data

To determine the gene expression patterns, we performed RNA-seq analysis using the *C. melo* reference genome. After filtering out the rRNAs and low-quality reads, 111 million reads were mapped to the reference genome (Table S1). For these clean reads, we obtained an average mapped read per sample of greater than 90%. One exception was the low (74.44%) alignment ratio obtained for Yaolong at 40 DAP. In total, we detected 21,172 expressed genes in the melon fruit samples.

**Fig. 3:**
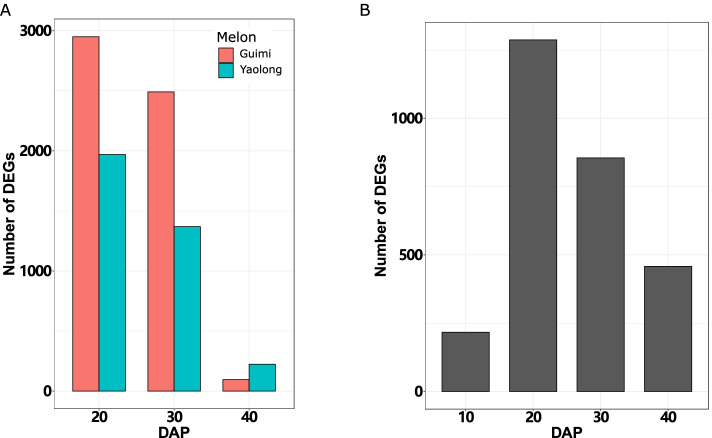
Distribution of differentially expressed genes (DEGs) at different time points after pollination during melon fruit development and ripening. (**A**) The number of DEGs in different samples. Light coral and medium turquoise colors denote DEGs in the Guimi and Yaolong varieties, respectively. (**B**) Distribution of DEGs between Guimi and Yaolong at specific sampling times

To determine the differences in the expression of genes between two close sampling times, we identified the DEGs in Guimi and Yaolong based on the threshold criteria of a log2 fold change ≥ 1 and FDR ≤ 0.05. For this purpose, we defined the earliest time point (10 DAP) of the paired groups as the control sample for subsequent measurements. The number of DEGs in Guimi was found to be markedly higher than that in Yaolong at 20 and 30 DAP, whereas fewer DEGs were identified in Guimi than Yaolong (223 vs. 95) at 40 DAP (Fig. 3A, Table S2). These findings indicate that during fruit development, the constituents of Guimi fruit undergo more pronounced changes than those of Yaolong fruit. However, over time, there was a gradual reduction in the number of DEGs in both melon varieties during fruit development, indicating a corresponding reduction in the speed of fruit growth and that the fruit was fully mature at 40 DAP. For both Guimi and Yaolong, we detected perturbations in gene expression at the same time points. The number of genes differentially expressed between Guimi and Yaolong initially increased, reaching a peak at 20 DAP, after which the number declined to 458 at 40 DAP (Fig. 3B). The majority of the top 30 significantly altered genes were downregulated in the comparisons between Yaolong and Guimi at 10, 20, and 40 DAP (Table S3, S4, S5, S6). We observed that caffeoyl-CoA O-methyltransferase-like (At1g67980), which is involved in the reinforcement of the plant cell wall, was more highly expressed in Yaolong during fruit development compared with the expression in Guimi.

For functional annotation, the DEGs identified in the two melon varieties were assigned to GO terms and KEGG pathways. We found that for Yaolong melons, the majority of enriched GO terms between 10 and 20 DAP were involved in biological processes, including ‘histone lysine methylation,’ ‘peptidyl-lysine methylation,’ and ‘DNA alkylation,’ whereas for Guimi melons, the most significantly enriched GO terms were ‘polysaccharide metabolism,’ ‘cell wall biogenesis,’ and ‘external encapsulating structure organization’ (Fig. S1 and S2). For the second comparison (20 DAP vs. 30 DAP), the DEGs in Guimi melons were found to be significantly enriched in the processes of ‘response to acid chemical,’ ‘plant-type cell wall biogenesis,’ and ‘response to chemical,’ whereas for Yaolong, the three most enriched biological processes were ‘hemicellulose metabolism,’ xylan metabolism,’ and ‘cell wall polysaccharide metabolism.’ Comparisons between 30 and 40 DAP samples revealed that the DEGs of Guimi melons were notably enriched for ‘amine metabolism,’ ‘cellular amine metabolism,’ and ‘jasmonic acid metabolism,’ whereas those of Yaolong melons were enriched in the cellular component categories ‘external encapsulating structure,’ ‘cell periphery,’ and ‘vacuole.’ With regards to KEGG pathway annotation, we found that in the 10 to 20 DAP comparison, DEGs in Yaolong were enriched for ‘DNA replication,’ ‘ABC transporters,’ and ‘flavone and flavonol biosynthesis,’ whereas ‘metabolic pathways,’ ‘biosynthesis of secondary metabolites,’ and ‘phenylpropanoid biosynthesis’ were enriched by Guimi DEGs (Fig. S3 and S4). Interestingly, for the 20 to 30 DAP comparison, we observed that for both melon varieties, the three most significantly DEG-enriched KEGG pathways were ‘biosynthesis of secondary metabolites,’ ‘metabolic pathways,’ and ‘phenylpropanoid biosynthesis.’ For the third comparison between 30 and 40 DAP, Guimi DEGs were found to be enriched in pathways such as ‘valine, leucine, and isoleucine biosynthesis,’ ‘biosynthesis of secondary metabolites,’ and ‘cyanoamino acid metabolism,’ whereas pathways enriched with Yaolong DEGs included ‘biosynthesis of secondary metabolites’ and ‘metabolic pathways.’

### Comparison of trends in temporal gene expression during melon fruit ripening

**Fig. 4:**
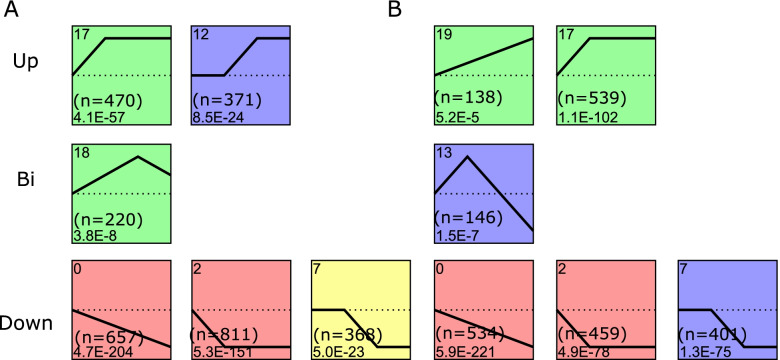
Enriched profiles of differentially expressed genes (DEGs) during fruit development. Profiles of Guimi (**A**) and Yaolong (**B**) were clustered into three groups, namely Up (upregulated), Bi (biphasic expression pattern), and Down (downregulated). Profile numbers are indicated in the top left-hand corner, and the corresponding *P*-values for each profile are shown in the bottom left-hand corner. The number of DEGs within each profile is shown in brackets

To gain further insight into the changes in gene expression during fruit development, we clustered 4,731 DEGs from Guimi melons and 3,198 DEGs from Yaolong melons into 38 profiles using the STEM algorithm. Among these, 2,897 Guimi DEGs were significantly clustered into the following six profiles: two upregulated profiles (profiles 17 and 12), three downregulated profiles (profiles 0, 7, and 2), and one biphasic expression pattern profile (profile 18) (Fig. 4A). Similarly, 2,217 Yaolong DEGs were classified into the following six profiles based on *P-*values ≤ 0.05: two upregulated patterns, one biphasic expression pattern, and three downregulated patterns (Fig. 4B).

To systematically investigate the biological functions of candidate genes, we extracted DEGs from the up- and downregulated cluster groups for further GO term and KEGG pathway analyses. GO analysis revealed 18 biological processes significantly enriched by the Yaolong DEGs assigned to profile 17 (Fig. S5A), and the DEGs in profile 19 were strongly categorized into two molecular processes (Fig. S5B), whereas the Guimi DEGs in profile 12 were enriched with respect to 110 major functions in the biological process, cellular component, and molecular function categories (Fig. S5C). However, we detected no significant enrichment of the Guimi DEGs clustered in profile 17 based on the adjusted *P*-values (Fig. S5D). The GO terms with the highest representation for the downregulated cluster groups are shown in Fig. S6. Among biological functions, ‘gibberellin metabolic process’ (GO:0,009,685), ‘cytoskeleton’ (GO:0,005,856), and ‘cell wall organization or biogenesis’ (GO:0,071,554) were the most significantly enriched functions in Guimi profiles 7, 2, and 0, respectively. For profile 0, 2, and 7 of Yaolong DEGs, ‘cell wall’ (GO:0,005,618), ‘histone lysine methylation’ (GO:0,034,968), and ‘phenylpropanoid biosynthetic process’ (GO:0,009,699), respectively, were the most enriched biological functions.

Based on KEGG analysis, we identified that nine KEGG pathways including ‘metabolic pathways’ (ko01100), ‘biosynthesis of secondary metabolites’ (ko01110), ‘phenylpropanoid biosynthesis’ (ko00940), and ‘biosynthesis of various secondary metabolites—part 2’ (ko00998) were enriched with Guimi DEGs assigned to downregulated profile 0, 2, and 7 (Fig. S7A). In contrast, only two pathways were enriched with Guimi DEGs within profiles 12 and 17, namely ‘carbon fixation in photosynthetic organisms’ (ko00710) and ‘galactose metabolism’ (ko00052) (Fig. S8A). In total, we identified nine pathways including ‘phenylpropanoid biosynthesis’ (ko0094), ‘metabolic pathways’ (ko01100), ‘biosynthesis of secondary metabolites’ (ko01110), ‘phagosome’ (ko04145), ‘purine metabolism’ (ko00230), ‘starch and sucrose metabolism’ (ko00500), ‘DNA replication’ (ko03030), ‘phenylalanine, tyrosine, and tryptophan biosynthesis’ (ko00400), and ‘biosynthesis of amino acids’ (ko01230) with significant enrichment of downregulated Yaolong DEGs (Fig. S7B). Protein processing in the endoplasmic reticulum (ko04141) was highly enriched by Yaolong DEGs in profiles 19 and 17 (Fig. S8B). These results indicate that most DEGs regulated during fruit development appear to be associated with the functioning of metabolic pathways.

### Analysis of cell wall biogenesis during melon fruit ripening

**Fig. 5:**
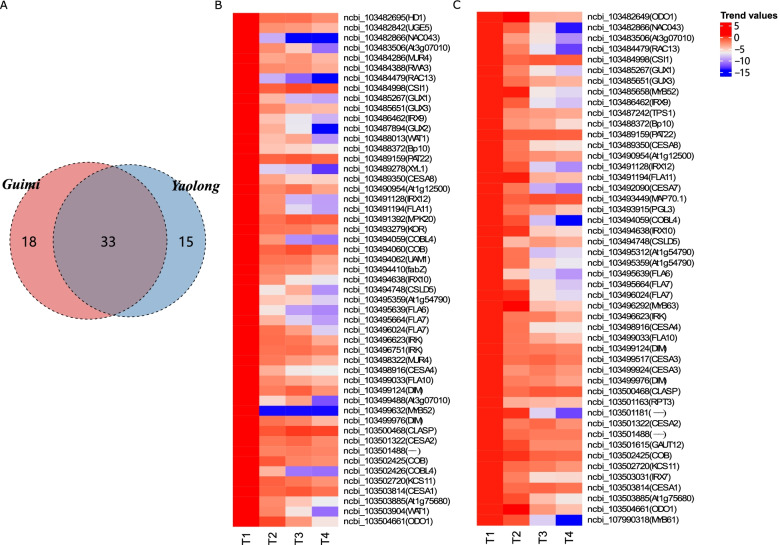
Trends in the expression changes of key genes associated with cell wall biogenesis in two Hami melon varieties. (**A**) Common differentially expressed genes (DEGs) in the cell wall biogenesis pathway of the two varieties. DEGs for Guimi (**B**) and Yaolong (**C**)

Fifty-one DEGs in Guimi, clustered in profiles 0 (*n* = 28) and 2 (*n* = 23), were significantly associated with cell wall biogenesis, showing downregulated gene expression patterns; 48 DEGs showing downregulated trends, clustered into profiles 0, 2, and 7, showed similar associations. Figure 5 shows the differences in the expression trends of these DEGs between Guimi and Yaolong. We observed 33 common DEGs in the cell wall biogenesis-related profiles of both melon varieties, the majority of which showed similar patterns of expression regulation at different stages of fruit development and ripening (Fig. 5A). For example, gradual reductions in GUX3 expression levels were observed during fruit development in both germplasms (Fig. 5B and C). A similar reduction in expression was detected for ODO1, which peaked during the early stages of fruit development, after which there was a slight reduction that became more pronounced prior to maturity. Although melon type-specific DEGs involved in cell wall biogenesis showed an overall downregulated pattern, changes in the direction of gene expression were still different between consecutive stages of development. For example, in Guimi melons, UAM1 was downregulated from stages 1 to 2, and then constantly expressed at a stable level from stages 2 to 3, prior to undergoing a decline in the mature stages. These findings indicated differences in the expression patterns of melon cultivar-specific DEGs associated with cell wall synthesis during fruit development.

### Analysis of sugar metabolism during melon fruit ripening

**Fig. 6:**
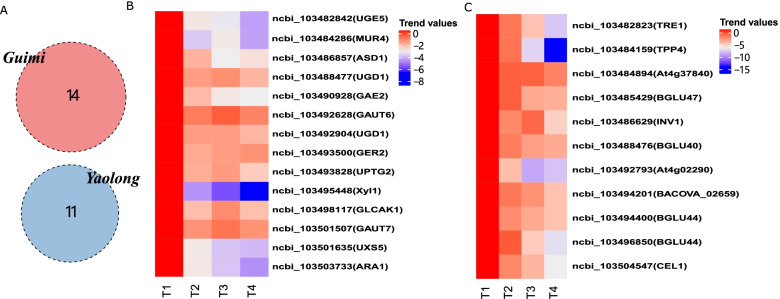
Trends in the expression changes of key genes associated with sugar metabolism pathways in the two Hami melon varieties. (**A**) The number of differentially expressed genes (DEGs) in sugar metabolism pathways in the two varieties. Expression trends of DEGs in Guimi (**B**) and Yaolong (**C**)

Some of the genes involved in sugar metabolism were found to be differentially expressed during melon fruit development, including those associated with ‘galactose metabolism,’ ‘starch and sucrose metabolism,’ ‘fructose and mannose metabolism,’ and ‘amino sugar and nucleotide sugar metabolism’ (Fig. 6A). However, in the case of Yaolong, only profile 0 DEGs were significantly associated with starch and sucrose metabolism pathways. In contrast, in Guimi, ‘amino sugar and nucleotide sugar metabolism’ were enriched with profile 2 DEGs. For the majority of DEGs associated with the ‘amino sugar and nucleotide sugar metabolism’ pathways, including MUR4, UGD1, and UPTG2, the lowest levels of expression were detected in ripe fruit (Fig. 6B). Differences in gene expression were also detected during different stages of fruit development. For example, there was a marked reduction in the expression of GAUT6 during the middle phase of fruit development, followed by a slight increase in pre-mature fruit, before declining to the lowest levels in mature fruit. Although the trend in UGD1 expression in young fruit was similar to that observed for GAUT6, it differed in that it was expressed at a constant level in the pre-mature fruit. In Yaolong, a total of 11 profile 0 DEGs were significantly associated with starch and sucrose metabolism pathways. In contrast to trends in the expression of sugar metabolism-related genes in Guimi, nine of these DEGs were characterized by gradually downregulated expression during fruit development and ripening (Fig. 6C). For example, the lowest level of At4g02290 expression in Yaolong was detected at stage T3. Conversely, whereas INV1 was characterized by a downregulated expression trend in young fruit, high expression levels were detected during the expansion stage, prior to a subsequent decline in expression, reaching the lowest values in ripe fruit.

## Discussion

The changes in the physicochemical characteristics of Guimi and Yaolong melon during fruit development, including weight, size, and soluble sugar content (Fig. 2), seemed to influence the flavor, taste, storage, and processing of Hami melons [18]. Interestingly, we also found that fruit weight increased rapidly from 35 to 40 DAP, whereas there were only slight increases in size, indicating an increase in the moisture content of the fruit (Fig. 2). Moisture content contributes to indirect estimations of fruit maturity and quality as well as the content of soluble solids because moisture and soluble solids content are highly negatively correlated in melon fruit [19]. Fruit ripening is a complex, genetically programmed process, which has not been extensively studied in Hami melon with respect to the underlying molecular mechanisms. To gain a better understanding of the associated gene regulation, we performed RNA-seq analysis of the Guimi and Yaolong varieties, from which we identified 21,172 genes that were expressed among the melon fruit samples during different stages of development. Of these genes, the numbers of differentially expressed genes in Guimi were found to be markedly higher than those in Yaolong at 20, 30, and 40 DAP (Fig. 3), which indicates that Guimi fruit undergoes more pronounced changes during the course of development, which is assumed to be related to its improved characteristics compared with that of Yaolong.

In the present study, we identified polysaccharide metabolism, cell wall biogenesis, and external encapsulating structure organization as the most significantly enriched GO terms among the Guimi melon DEGs between 10 and 20 DAP (Fig. S1 and S2). Similarities in differential gene expression patterns between 10 and 20 DAP and 30 and 40 DAP during fruit development have been reported previously for *Cucumis melo*, indicating that gene expression is significantly altered from 20 to 30 DAP, with implications for fruit development [20]. Based on our observations, we speculate that the differences between Yaolong and Guimi, with respect to cell wall biogenesis, begin to manifest at an early point in fruit development. Moreover, in Guimi melons, we detected differences in the number of metabolic pathways, notably, ‘metabolic pathways,’ ‘biosynthesis of secondary metabolites,’ and ‘phenylpropanoid biosynthesis,’ between 10 and 20 DAP (Figs. S3 and S4), which is likely an indication of the early modification of metabolic regulation during the ripening stage. Plant polysaccharide metabolic pathways play a prominent role in mediating fruit ripening [21].

Melons are consumed for their sweet taste [22], a trait that can be attributed to the accumulation of sucrose during the late stage of fruit development [23–25], and in this context, we found that DEGs associated with plant polysaccharide metabolic pathways showed different gene expression patterns during the fruit ripening process, thereby implying a role in the regulation of fruit ripening.

In total, we detected 4,731 and 3,198 genes that were differentially expressed in the Guimi and Yaolong varieties, respectively, during fruit development, which were clustered into 38 profiles based on STEM analysis. Three downregulated profiles (0, 2, and 7) were obtained for both Guimi and Yaolong melons, whereas upregulated profiles were notably more prominent in Guimi melons (Fig. 4). In Guimi melons, we identified several pathways enriched with upregulated DEGs, namely, ‘carbon fixation in photosynthetic organisms’ and ‘galactose metabolism’ (Fig. S8), whereas no significantly enriched pathways were identified for the upregulated DEGs in the Yaolong melons. The upregulated activity of these pathways has been reported previously and is considered particularly advantageous for the breeding of Hami melons. Carbon fixation pathways are ubiquitous in plants, algae, cyanobacteria, and other photosynthetic organisms. High CO_2_ concentrations provide an abundant resource for plant photosynthesis and promote plant growth and biomass accumulation [26]. Yelle et al. found that in tomatoes, CO_2_ accelerated flower bud differentiation in fruit and vegetable crops, reduced the node position of female flowers, increased the number of female flowers and fruit setting rate, promoted fruit growth, and increased single fruit weight and early yield [27]. Similarly, Zhao et al. and Zhu et al. showed that increasing the application of CO_2_ promoted a significant increase in the growth of melon seedlings, along with significant increases in plant height, stem diameter, and leaf area, which are conducive to the cultivation of strong seedlings [28, 29]. The upregulated expression of carbon fixation pathway-associated genes in Guimi melons is assumed to enhance the photosynthetic efficiency of plants [30, 31] and promote the accumulation of organic matter and plant growth [32]. An increase in CO_2_ levels promotes an increase in H^+^ concentration in the cell wall, activates enzymes that soften the cell wall, releases the connection of polymers in cell walls, causes relaxation of the cell wall, and contributes to reductions in turgor pressure, thereby resulting in a thickening of leaves and an increase in leaf area [33]. Consequently, photosynthesis-mediated carbon fixation is important for fruit ripening.

Cell wall metabolism is one of the most important aspects of fruit ripening and is closely associated with flesh texture [34], which is a significant quality attribute defining commercial fruit quality [4]. Plant cell walls play a significant role in determining the shape and elasticity of fruits, and changes in cell wall structure and composition are considered primary factors contributing to changes in fruit texture [35]. Fruit texture is an important factor affecting fruit quality, storage and transportation characteristics, and disease resistance. Changes in pulp cell wall components are the primary factors contributing to changes in fruit essence [36], whereas fruit softening is positively correlated with the degradation of cell wall components caused by cell wall hydrolases [37]. Studies have shown that mutations in several key genes involved in plant hormone synthesis and signal transduction can lead to downregulation of cell wall-related genes. For example, the aldose content and the degree of pectin methyl esterification were found to be reduced in gibberellin gal-3 and Gai mutants [38]. The expression of xyloglucan endotransglucosylase hydrolase (XTH) in *Arabidopsis* hypocotyls is induced by gibberellin [39], and in the present study, we established that gibberellin metabolic process (GO:0,009,685), cytoskeleton (GO:0,005,856), and cell wall organization or biogenesis (GO:0,071,554) were the most significantly enriched functions in Guimi melon DEG profiles (Fig. 4). This indicates that the genes involved in cell wall metabolism might play essential roles in determining fruit ripening. Moreover, we found that GUX3 expression gradually decreased during fruit development. Gux, a glucuronosyltransferase-related gene, plays an important role in the formation of xylan side chains [40, 41]. In *Arabidopsis*, GUX3 is expressed in roots, stems, leaves, and flowers, although only in the xylem. Consequently, it may be involved in the synthesis of certain components of the protowall [42], however, its associated functions have not yet been reported.

Sugars are essential components of melon fruit quality and serve as prominent signals for the regulation of fruit ripening [43, 44]. The soluble sugars found in fruits are primarily sucrose, fructose, and glucose [45, 46], the accumulation of which is closely associated with fruit ripening. Sugars serve as regulatory signals for the expression of numerous genes, among which sucrose is considered the most effective signal molecule. For example, sucrose has been demonstrated to function as a signal molecule that interacts with abscisic acid to regulate strawberry fruit ripening [43]. In the present study, we identified several genes involved in sugar metabolism showing differential expression patterns during fruit development, including those associated with ‘galactose metabolism,’ ‘starch and sucrose metabolism,’ ‘fructose and mannose metabolism,’ and ‘amino sugar and nucleotide sugar metabolism’ (Fig. 6). In the case of several of these genes, we detected reduced expression levels during the middle phase of fruit development, followed by a slight increase in the pre-mature fruit stage, which subsequently declined to the lowest recorded levels of expression in the mature fruit (Fig. 6). The sugar content differs according to organ and tissue, and the types of sugar also vary; for example, the vascular bundles of tomato pedicels and fruit stalks mainly contain sucrose, whereas in other parts of the fruit, the accumulated sugars are primarily glucose and fructose. Moreover, the glucose and fructose contents in the pericarp tissue and vascular bundles of tomato fruit differ significantly from those in the pectin and septum [47]. Similarly, whereas the predominant sugars in grapes are glucose, peaches and apricots accumulate sucrose. These differences are generally determined by the type of sugar metabolism in the fruit and the activity of related enzymes [48]. It has also been established that there are considerable differences in sugar type and content among the different varieties of the same plant, as demonstrated in a study of nine white pear varieties [49]. In the early stages of fruit development, sucrose transporters Pu SUT and β-glucomannases Pu bglu1, Pu bglu2, and Pu bglu4 are highly expressed [50]. Numerous studies have examined the expression of the sucrose acid invertase gene. For example, Qin et al. characterized the cell wall acid invertase gene Mr ivr1 in *Myrica rubra*, and semi-quantitative RT-PCR expression analysis revealed its highest expression during the early stage of fruit development and relatively low expression in mature fruit [51]. Thus, consistent with the findings of the present study, it has been widely established that the expression of multiple genes associated with glucose metabolism differs among different species and varieties of fruit-bearing plants as well as at different stages of fruit ripening.

## Conclusion

In this study, we performed comparative transcriptomic analyses of two varieties of Hami melon (Guimi and Yaolong) with the aim of characterizing the genes and associated pathways controlling the physicochemical properties of fruits. We successfully identified several candidate genes and pathways associated with polysaccharide metabolism, cell wall processes, biosynthesis of secondary metabolites, phenylpropanoid biosynthesis, and carbon fixation, which probably contribute to the regulation of the size and flavor traits of these fruits. Comparing gene expression patterns at different stages of fruit development in Guimi melons will provide further insights into the regulatory mechanisms underlying the physicochemical characteristics of melon fruit during the progression of development.

## Methods

### Plant materials

The germplasm ‘Guimi’ was provided by the Vegetable Research Institute, Guangxi Academy of Agricultural Sciences, Nanning, China. The germplasm ‘Yaolong’ was purchased from Fuyou Seedlings Co. Ltd. (Hainan, China). We had got the permission to collect and use the two germplasm lines. The experimental research and field studies on plants comply with relevant institutional, national, and international guidelines and legislation. Both germplasms were cultivated in a greenhouse at daytime temperatures between 25–30 °C and above 15 °C at night. Flowers were manually pollinated, and three to five biological replicate fruits were harvested from selected plants at 10, 20, 30, and 40 DAP. These fruits were frozen in liquid nitrogen and stored at -80 °C until further analysis.

### Determination of physicochemical characteristics

Among the physical characteristics measured, the size and weight of fruit were measured at 5, 10, 15, 20, 25, 30, 35, and 40 DAP. The Brix value of the fruit was determined using an ATAGO PAL-2 refractometer as previously described [52]. Each measurement was conducted on at least three replicate fruits. The soluble sugar content (total soluble solids, glucose, fructose, and sucrose) was analyzed spectrophotometrically, as described previously [18].

### RNA isolation, cDNA library preparation, and RNA-Seq

Total RNA was extracted from the pulp of frozen fruit collected at each time point using a TRIzol reagent kit (Invitrogen, Carlsbad, CA, USA) according to the manufacturer’s protocol. The quality of the isolated total RNA was evaluated using an Agilent 2100 Bioanalyzer (Agilent Technologies, Palo Alto, CA, USA) and RNase-free agarose gel electrophoresis. mRNA was enriched, fragmented, and reverse-transcribed to first-strand cDNA using oligo (dT), fragmentation buffer, and random primers. Second-strand cDNA was synthesized using DNA polymerase I, RNase H, dNTPs, and a buffer. Finally, 24 cDNA libraries, including three replicates for each group, were constructed and sequenced using the Illumina Novaseq6000 platform with pair-end 150 bp mode.

### Global and differential gene expression analysis

To obtain high-quality clean reads, reads containing adapter and poly-N sequences or low-quality bases were filtered using fastp (version 0.18.0) [53] with default parameters. Thereafter, rRNA mapped reads were removed following the alignment of clean reads to a ribosomal RNA (rRNA) database using Bowtie2 (version 2.28) [54]. As a *Cucumis melo* reference genome, we used the RefSeq assembly accession number GCF_000313046.1. The remaining cleaned reads were aligned to the reference genome with HISAT2 (version 2.4) using “-rna-strandness RF” [55], and genes were assembled using StringTie (version 1.3.1) based on these mapped reads [56]. RSEM software was used to calculate gene expression values based on well-mapped reads and normalized to the fragments per kilobase of exon per million mapped fragment (FPKM) values, as previously described [57].

For analysis of differential gene expression between the Yaolong and Guimi groups, we used DESeq2 software. Differentially expressed genes (DEGs) were identified based on the criteria of a false discovery rate (FDR) < 0.05 and an absolute value of log2 (fold change) ≥ 1.

### Temporal analysis

To determine trends in the changes in gene expression during *C. melo* fruit development, we performed temporal analysis of the DEGs of each variety using Short Time-series Expression Miner (STEM) software with default parameters [58]. Genes were profiled by clustering based on the corresponding *P*-values and those with a *P*-value ≤ 0.05 were considered differentially expressed. These DEGs were subjected to GO term and KEGG pathway enrichment analyses using hypergeometric distribution tests. GO terms and KEGG pathways with Q-values ≤ 0.05 were considered functional annotations.

## Supplementary Information

**Additional file 1.**  

**Additional file 2.** 

**Additional file 3.** 

**Additional file 4.** 

**Additional file 5.** 

**Additional file 6.** 

**Additional file 7.** 

**Additional file 8.** 

**Additional file 9.** 

## Data Availability

The datasets used and/or analysed during the current study are available in the NCBI Bioproject repository, [PRJNA787394].

## Abbreviations

DEGs: Differentially expressed genes
DAP: Days after pollination
KEGG: Kyoto Encyclopedia of Genes and Genomes
GO: Gene Ontology
STEM: Short time-series expression miner
