# Supplementary material for: Comparative transcriptomic analysis of two Cucumis melo var. saccharinus germplasms differing in fruit physical and chemical characteristics
Source: BMC Plant Biol. 2022 Apr 12;22:193. doi: 10.1186/s12870-022-03550-8 (PMC9004126; doi:10.1186/s12870-022-03550-8)
Supplement: Supplementary file 1 — Additional file 1. [file 12870_2022_3550_MOESM1_ESM.pdf]

A

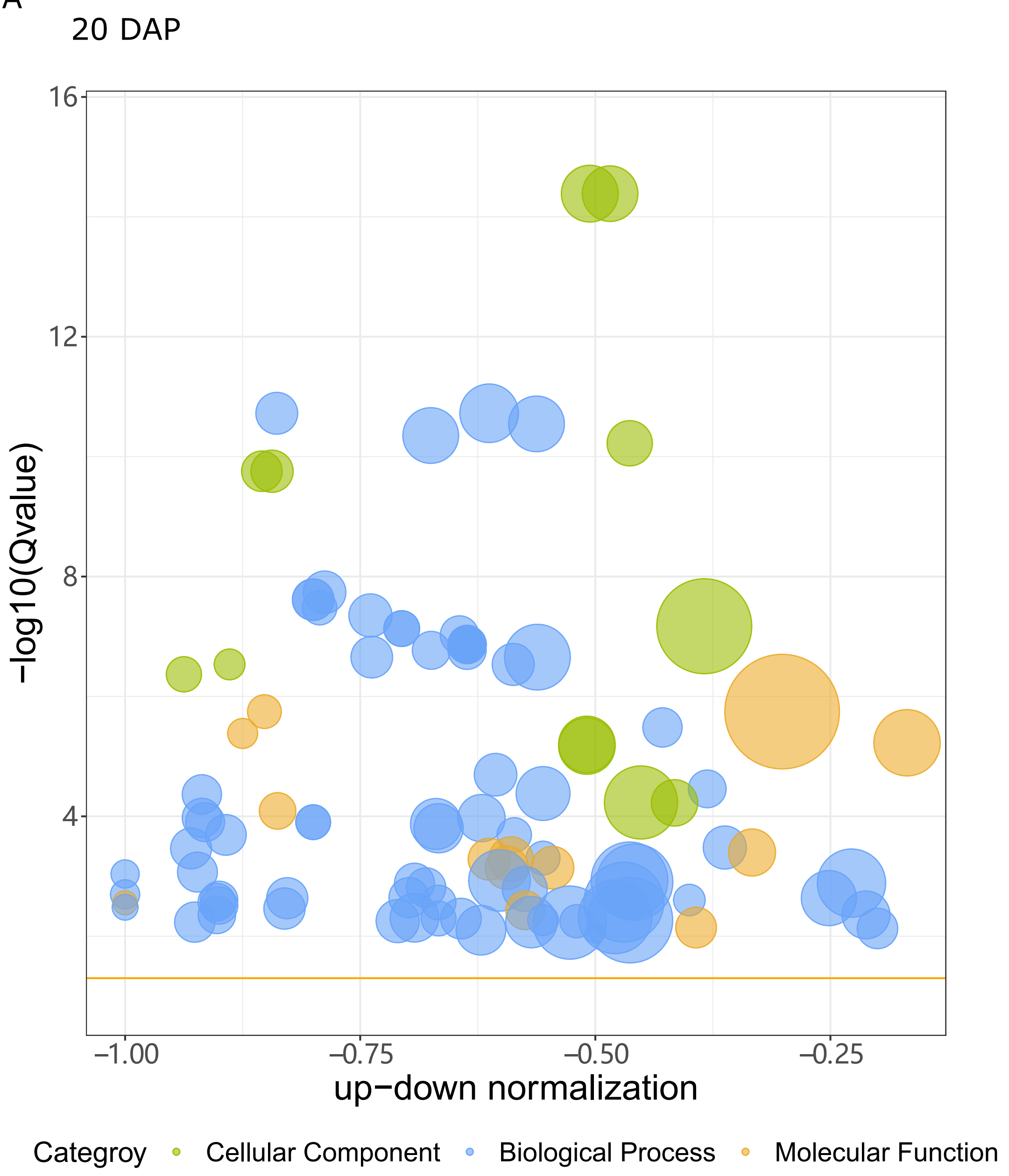

GO term Top 20

ID	Descrption
GO:0005976	polysaccharide metabolic process
GO:0042546	cell wall biogenesis
GO:0045229	external encapsulating structure organization
GO:0071554	cell wall organization or biogenesis
GO:0010383	cell wall polysaccharide metabolic process
GO:0010410	hemicellulose metabolic process
GO:0045491	xylan metabolic process
GO:0009832	plant−type cell wall biogenesis
GO:0044036	cell wall macromolecule metabolic process
GO:0032535	regulation of cellular component size
GO:0090066	regulation of anatomical structure size
GO:0060560	developmental growth involved in morphogenesis
GO:0043476	pigment accumulation
GO:0043478	pigment accumulation in response to UV light
GO:0071944	cell periphery
GO:0030312	external encapsulating structure
GO:0005618	cell wall
GO:0005856	cytoskeleton
GO:0015630	microtubule cytoskeleton
GO:0016020	membrane

B

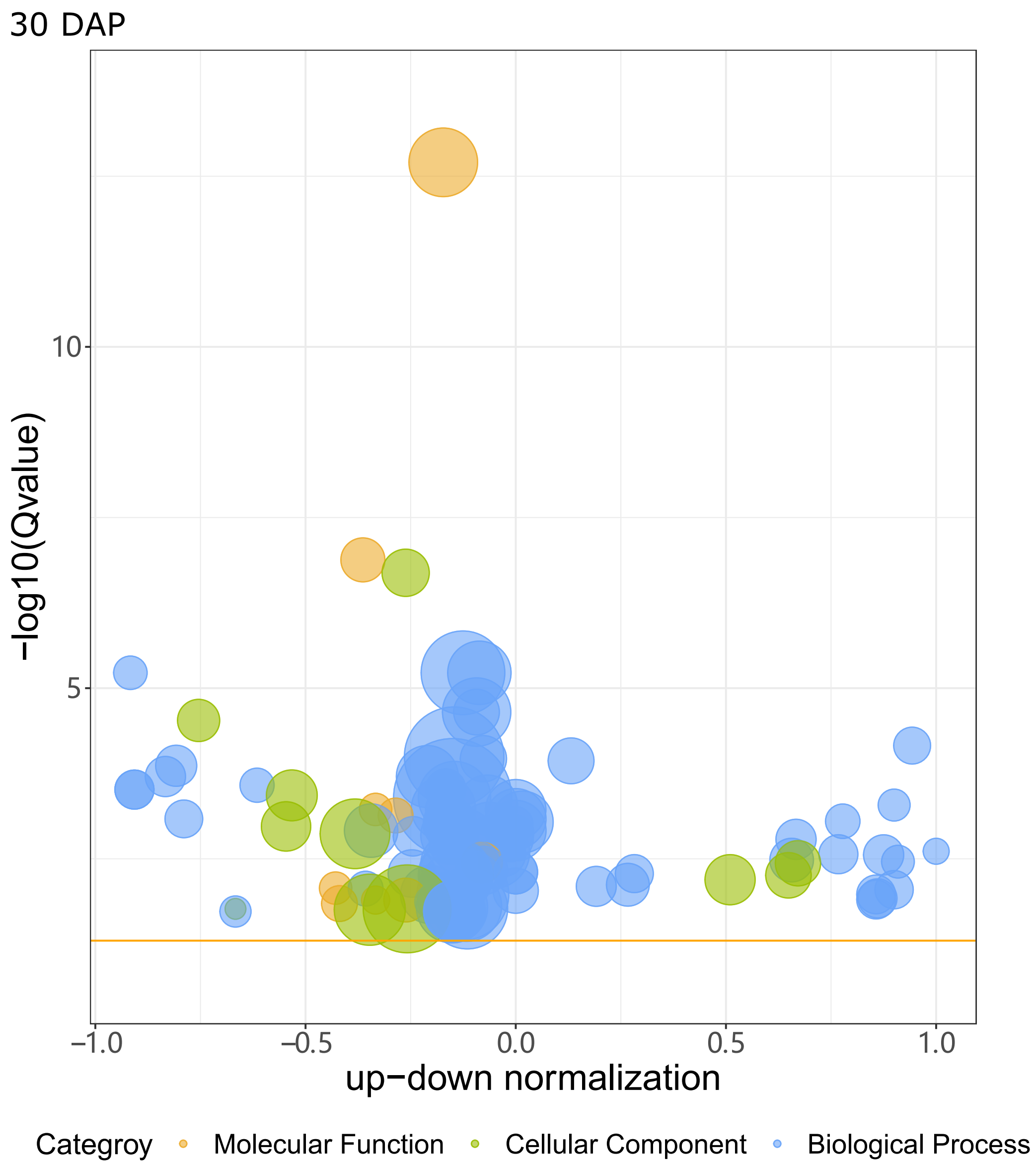

GO term Top 20

ID	Descrption
GO:0001101	response to acid chemical
GO:0009832	plant−type cell wall biogenesis
GO:0042221	response to chemical
GO:0006721	terpenoid metabolic process
GO:0009605	response to external stimulus
GO:0009658	chloroplast organization
GO:0050896	response to stimulus
GO:0006720	isoprenoid metabolic process
GO:0009620	response to fungus
GO:0044036	cell wall macromolecule metabolic process
GO:0010383	cell wall polysaccharide metabolic process
GO:0009725	response to hormone
GO:0010087	phloem or xylem histogenesis
GO:0009267	cellular response to starvation
GO:0042594	response to starvation
GO:0010410	hemicellulose metabolic process
GO:0005576	extracellular region
GO:0005618	cell wall
GO:0016491	oxidoreductase activity
GO:0016705	oxidoreductase activity, acting on paired donors, with incorporation or reduction of molecular oxygen

C

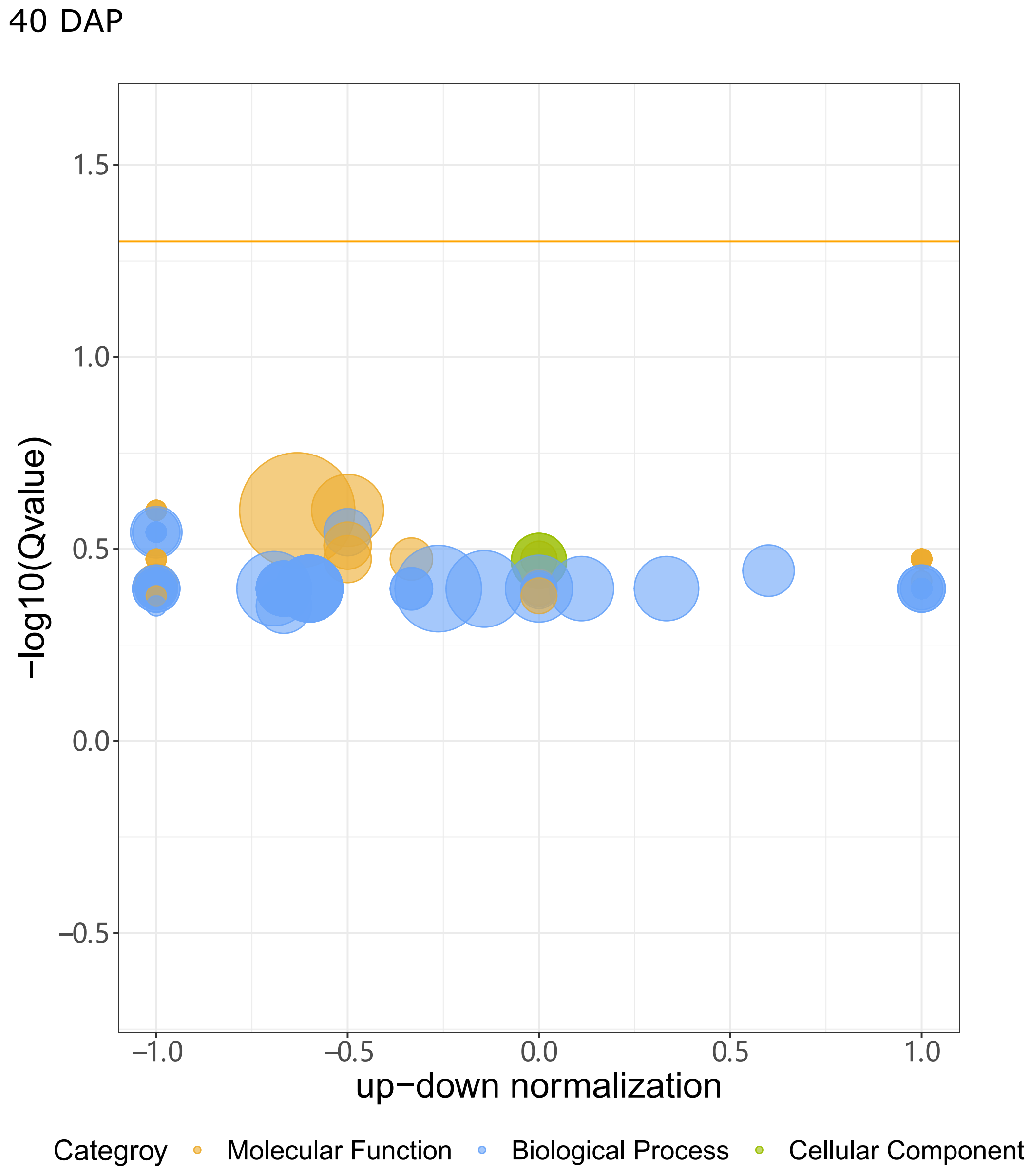

GO term Top 20

ID	Descrption
GO:0009308	amine metabolic process
GO:0044106	cellular amine metabolic process
GO:0009694	jasmonic acid metabolic process
GO:0001676	long−chain fatty acid metabolic process
GO:0006690	icosanoid metabolic process
GO:0019369	arachidonic acid metabolic process
GO:1901568	fatty acid derivative metabolic process
GO:0003824	catalytic activity
GO:0001948	glycoprotein binding
GO:0003867	4−aminobutyrate transaminase activity
GO:0015098	molybdate ion transmembrane transporter activity
GO:0016491	oxidoreductase activity
GO:0016614	oxidoreductase activity, acting on CH−OH group of donors
GO:0061135	endopeptidase regulator activity
GO:0046992	oxidoreductase activity, acting on X−H and Y−H to form an X−Y bond
GO:0046993	oxidoreductase activity, acting on X−H and Y−H to form an X−Y bond, with oxygen as acceptor
GO:0016616	oxidoreductase activity, acting on the CH−OH group of donors, NAD or NADP as acceptor
GO:0061134	peptidase regulator activity
GO:0016671	oxidoreductase activity, acting on a sulfur group of donors, disulfide as acceptor
GO:0016879	ligase activity, forming carbon−nitrogen bonds
