# Supplementary material for: Comparative transcriptomic analysis of two Cucumis melo var. saccharinus germplasms differing in fruit physical and chemical characteristics
Source: BMC Plant Biol. 2022 Apr 12;22:193. doi: 10.1186/s12870-022-03550-8 (PMC9004126; doi:10.1186/s12870-022-03550-8)
Supplement: Supplementary file 2 — Additional file 2. [file 12870_2022_3550_MOESM2_ESM.pdf]

A

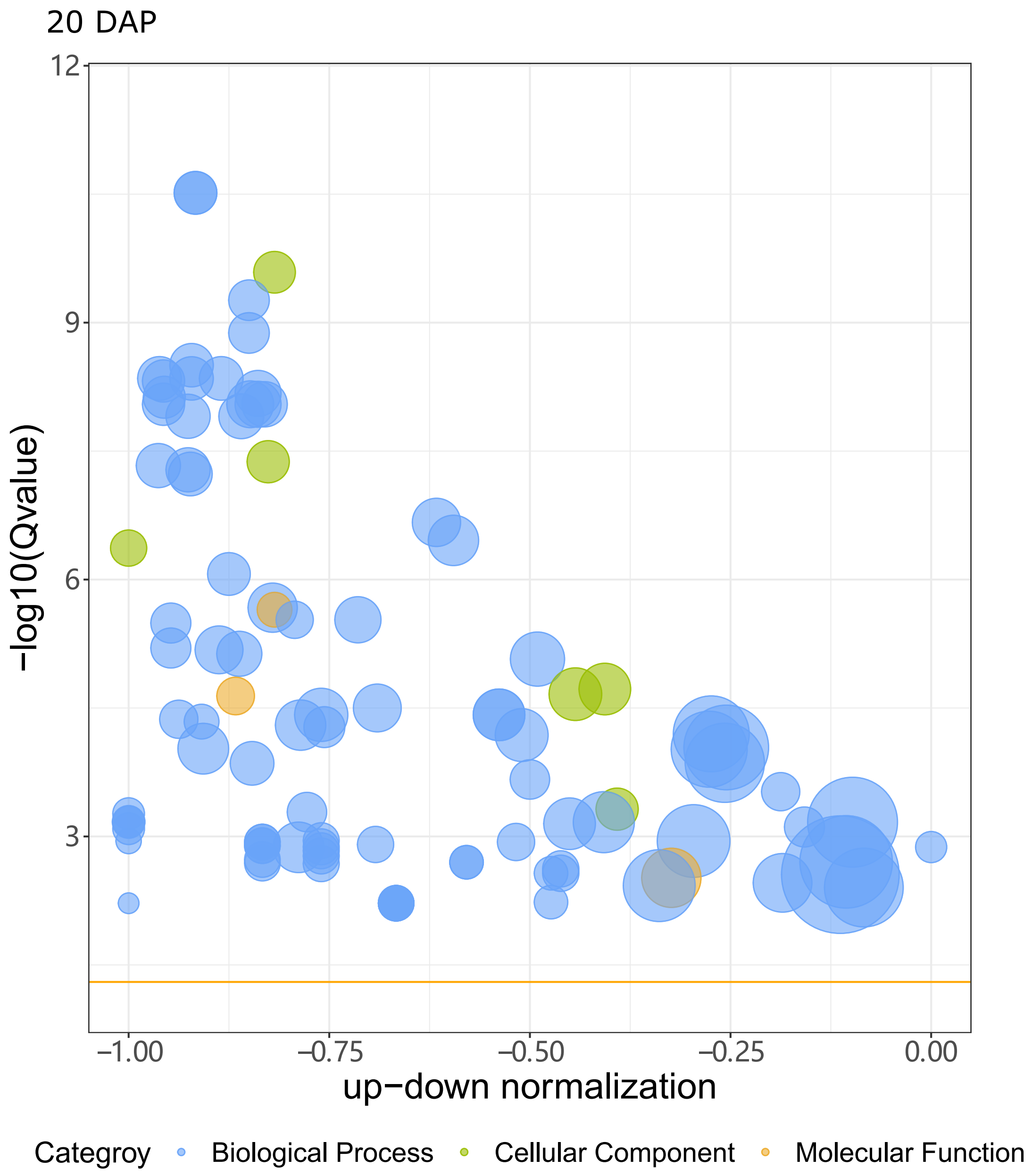

GO term Top 20

ID	Descrption
GO:0034968	histone lysine methylation
GO:0018022	peptidyl−lysine methylation
GO:0006305	DNA alkylation
GO:0006304	DNA modification
GO:0016571	histone methylation
GO:0008213	protein alkylation
GO:1903047	mitotic cell cycle process
GO:0006479	protein methylation
GO:0061640	cytoskeleton−dependent cytokinesis
GO:0016570	histone modification
GO:0000281	mitotic cytokinesis
GO:0018193	peptidyl−amino acid modification
GO:0000910	cytokinesis
GO:0016569	covalent chromatin modification
GO:0016568	chromatin modification
GO:0018205	peptidyl−lysine modification
GO:0043414	macromolecule methylation
GO:0000278	mitotic cell cycle
GO:0015630	microtubule cytoskeleton
GO:0005856	cytoskeleton

B

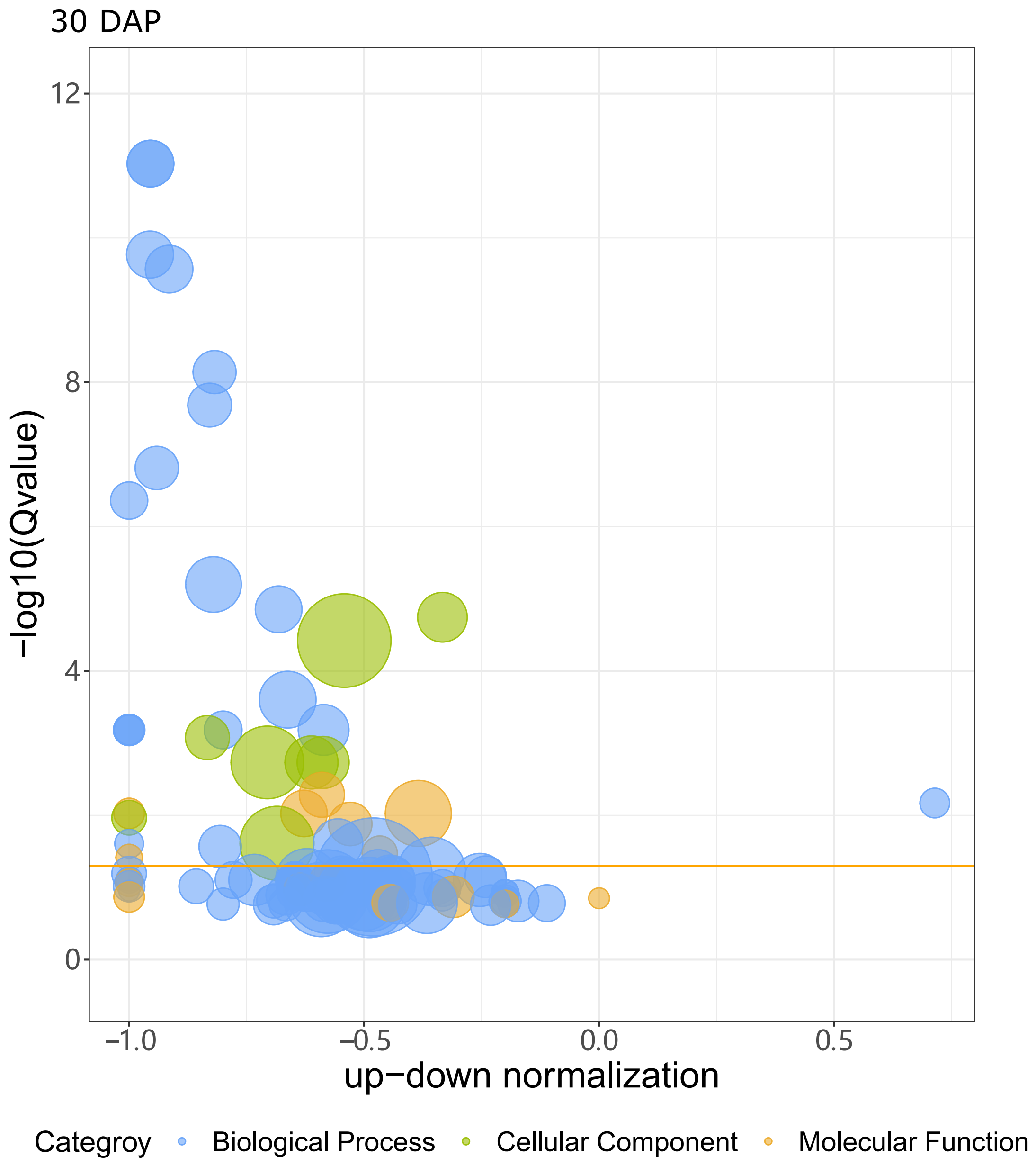

GO term Top 20

ID	Descrption
GO:0010410	hemicellulose metabolic process
GO:0045491	xylan metabolic process
GO:0010383	cell wall polysaccharide metabolic process
GO:0044036	cell wall macromolecule metabolic process
GO:0009699	phenylpropanoid biosynthetic process
GO:0009698	phenylpropanoid metabolic process
GO:0042546	cell wall biogenesis
GO:0009832	plant−type cell wall biogenesis
GO:0071554	cell wall organization or biogenesis
GO:0044550	secondary metabolite biosynthetic process
GO:0005976	polysaccharide metabolic process
GO:0010413	glucuronoxylan metabolic process
GO:0019748	secondary metabolic process
GO:0009891	positive regulation of biosynthetic process
GO:0006558	L−phenylalanine metabolic process
GO:1902221	erythrose 4−phosphate/phosphoenolpyruvate family amino acid metabolic process
GO:0005576	extracellular region
GO:0016020	membrane
GO:0005618	cell wall
GO:0030312	external encapsulating structure

C

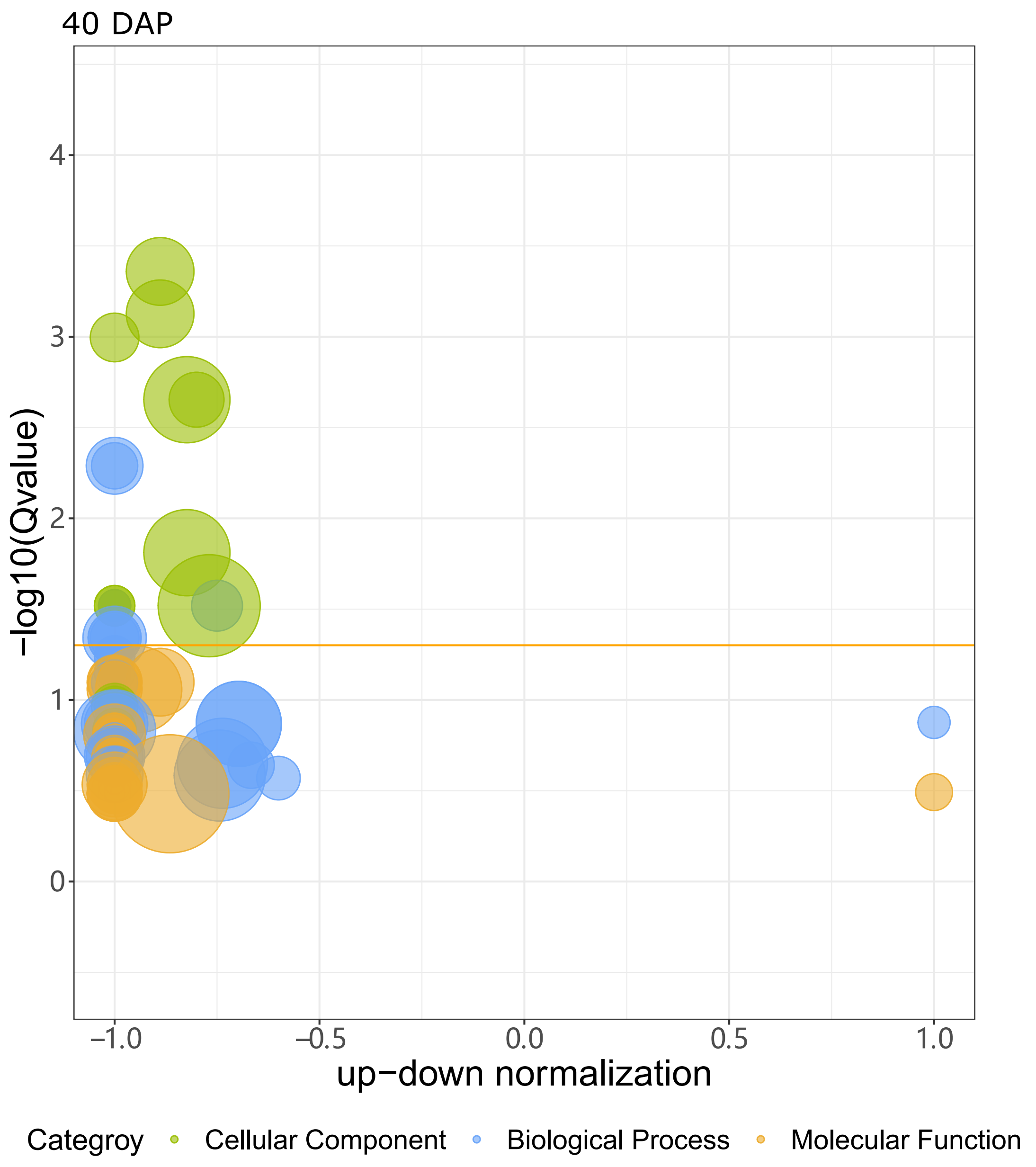

GO term Top 20

ID	Descrption
GO:0009832	plant−type cell wall biogenesis
GO:0071669	plant−type cell wall organization or biogenesis
GO:0033240	positive regulation of cellular amine metabolic process
GO:0045764	positive regulation of cellular amino acid metabolic process
GO:0009308	amine metabolic process
GO:0071554	cell wall organization or biogenesis
GO:0042546	cell wall biogenesis
GO:0022622	root system development
GO:0048364	root development
GO:0030198	extracellular matrix organization
GO:0043062	extracellular structure organization
GO:0030312	external encapsulating structure
GO:0071944	cell periphery
GO:0005773	vacuole
GO:0005618	cell wall
GO:0031224	intrinsic component of membrane
GO:0044425	membrane part
GO:0005774	vacuolar membrane
GO:0044437	vacuolar part
GO:0016020	membrane
