# Supplementary figures and images for: Comparative transcriptomic analysis of two Cucumis melo var. saccharinus germplasms differing in fruit physical and chemical characteristics
Source: BMC Plant Biol. 2022 Apr 12;22:193. doi: 10.1186/s12870-022-03550-8 (PMC9004126; doi:10.1186/s12870-022-03550-8)

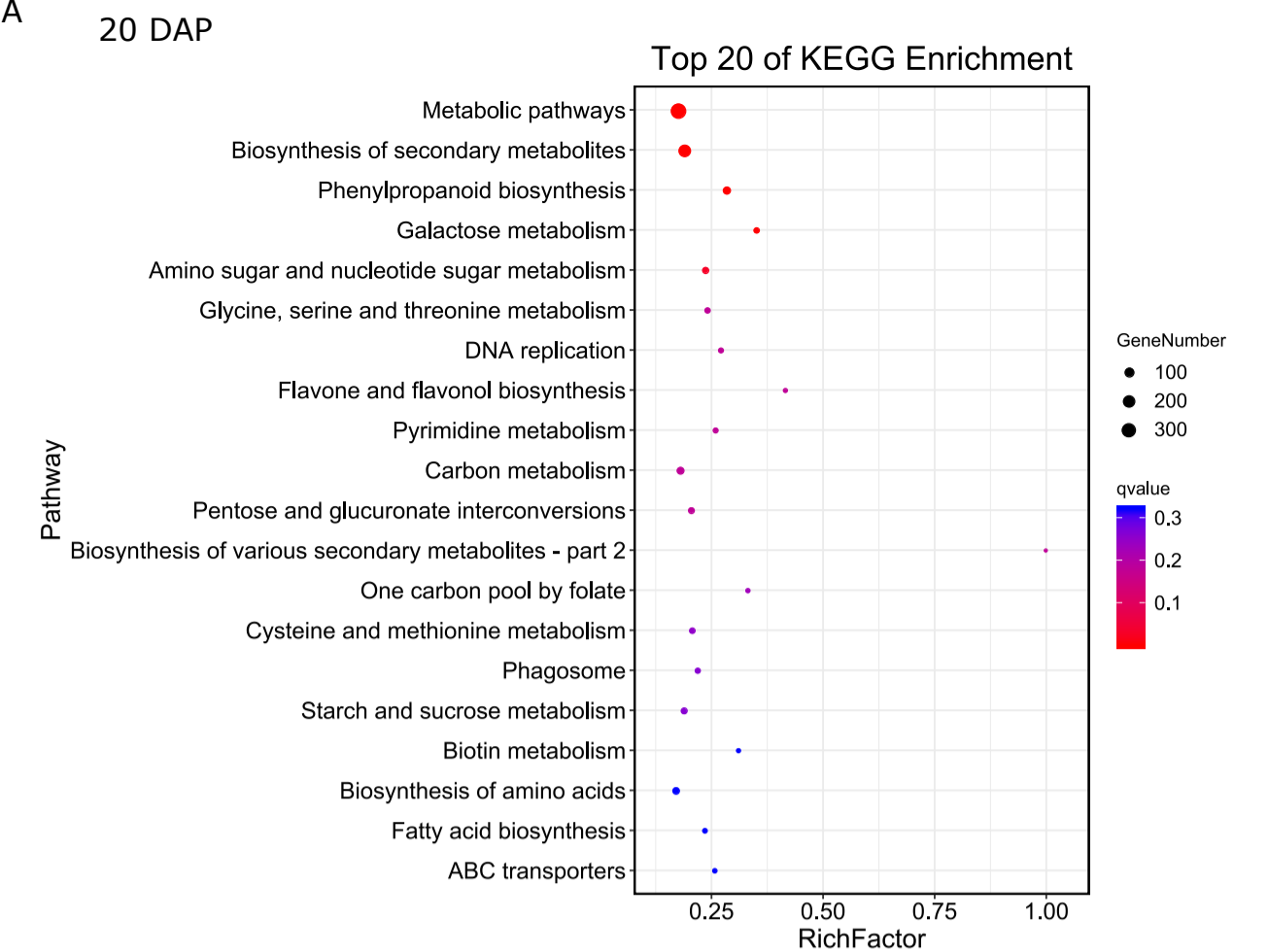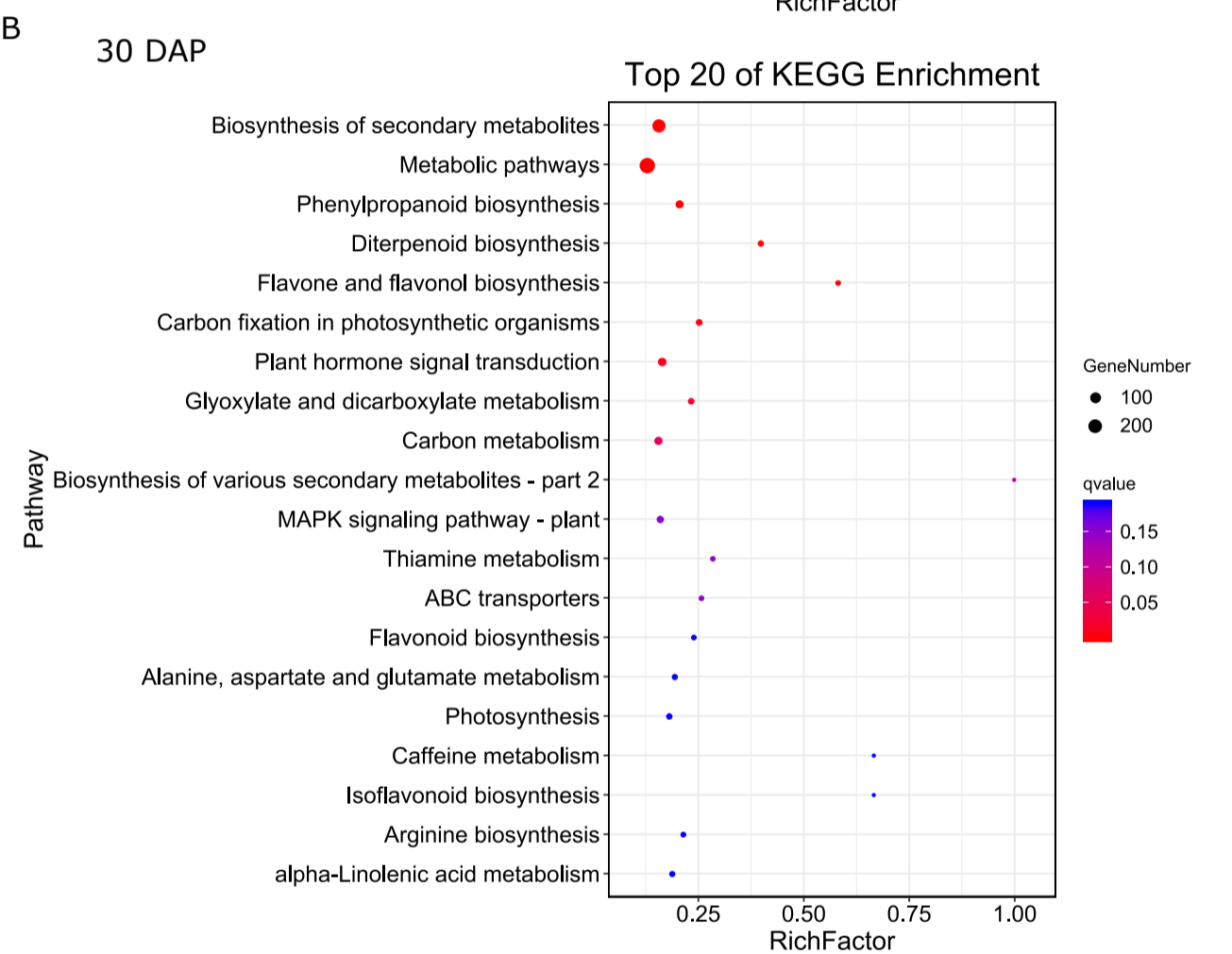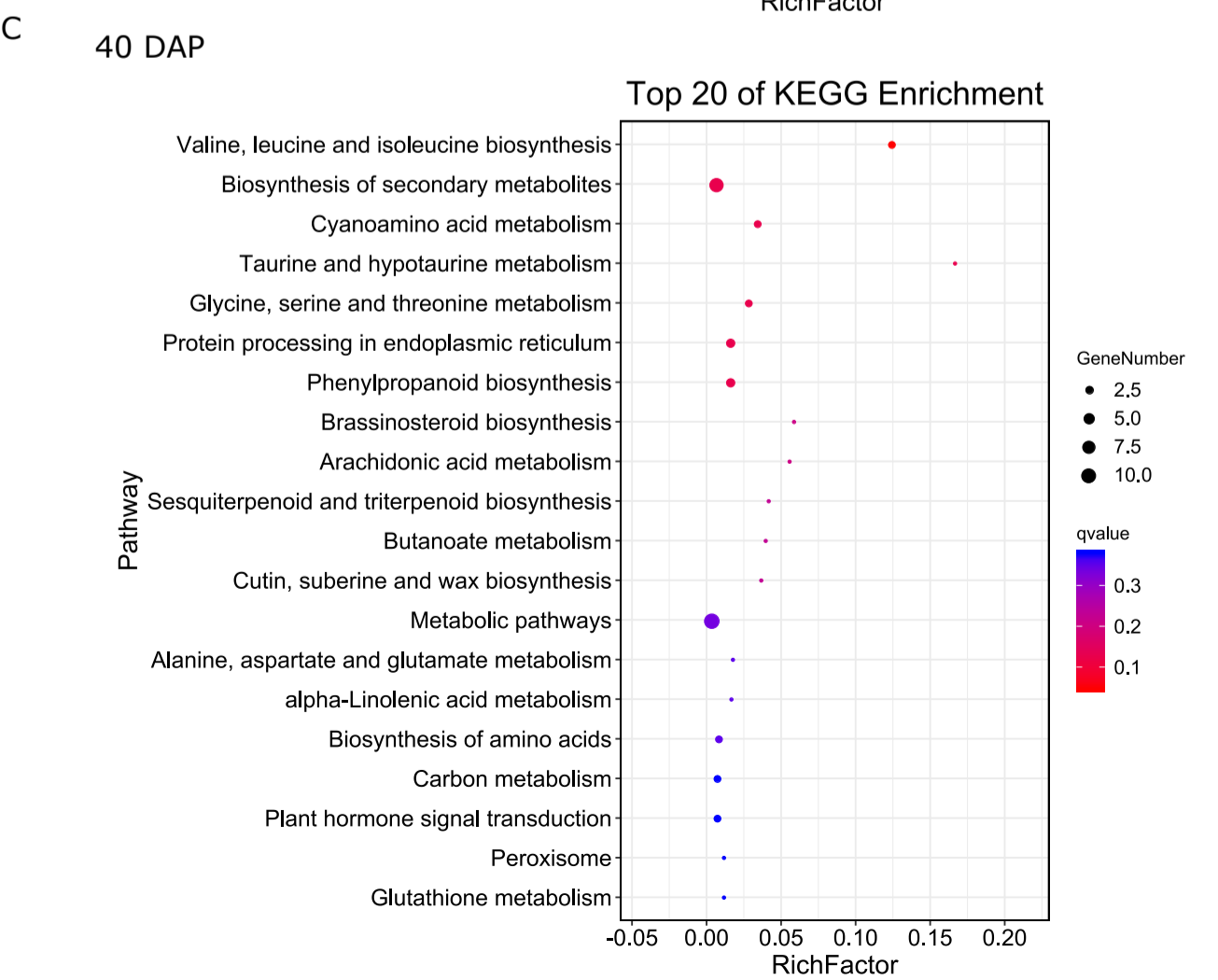

Supplement: Supplementary file 3 — Additional file 3. [file 12870_2022_3550_MOESM3_ESM.pdf]

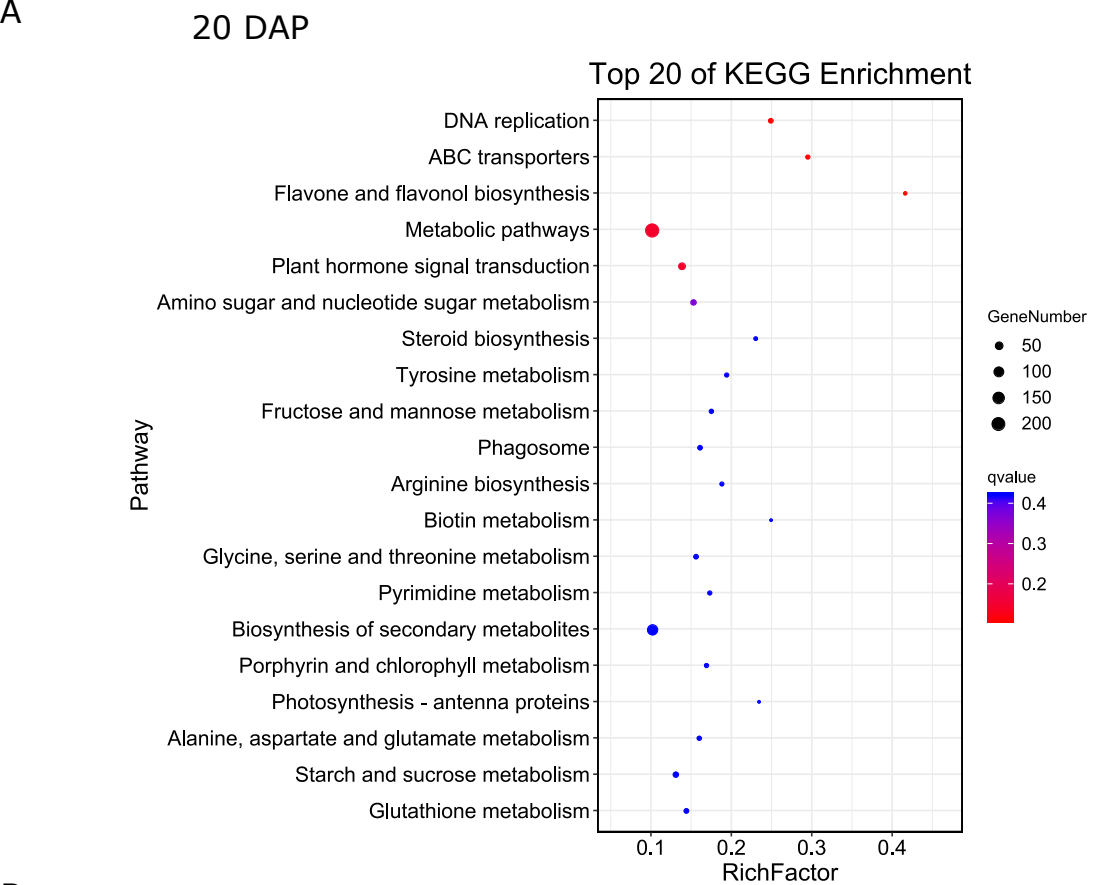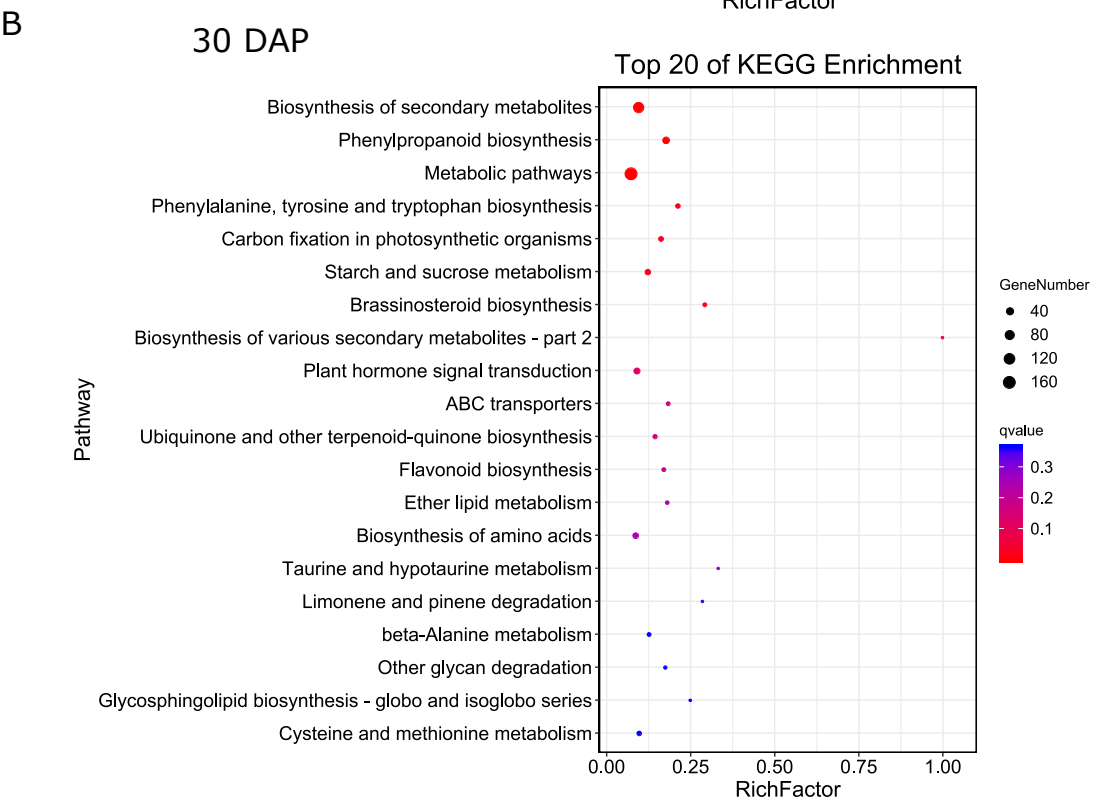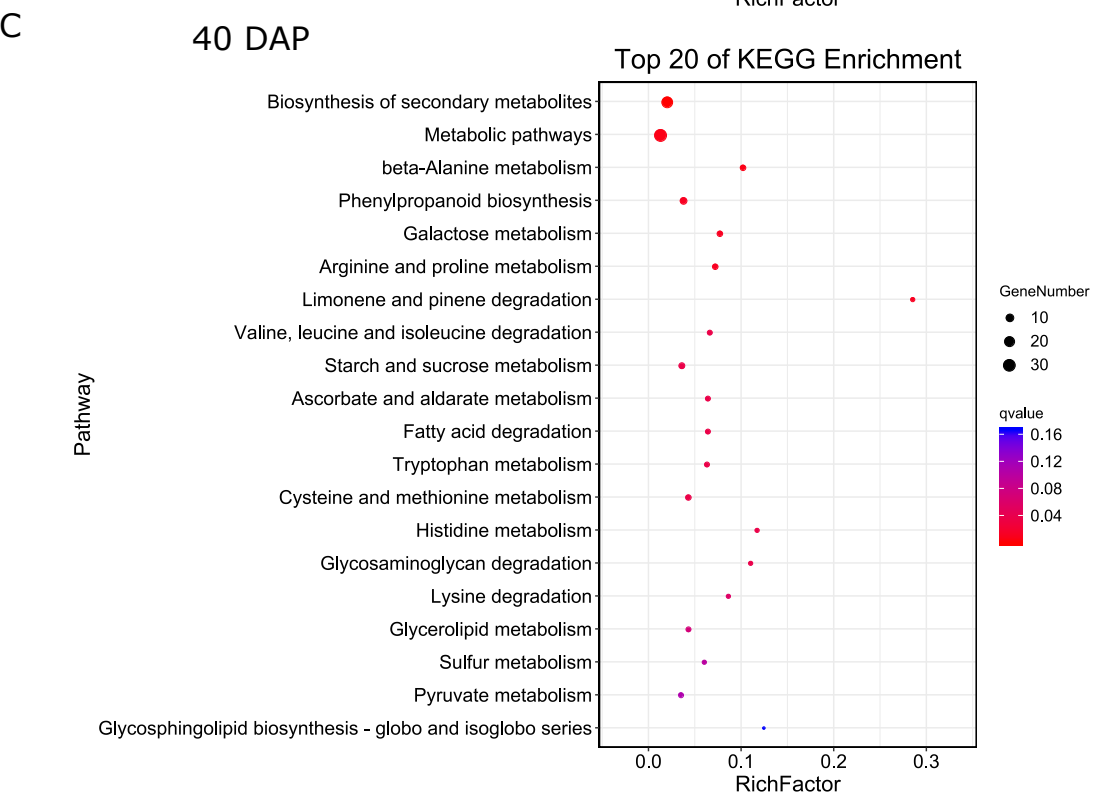

Supplement: Supplementary file 4 — Additional file 4. [file 12870_2022_3550_MOESM4_ESM.pdf]

A

Profile 17

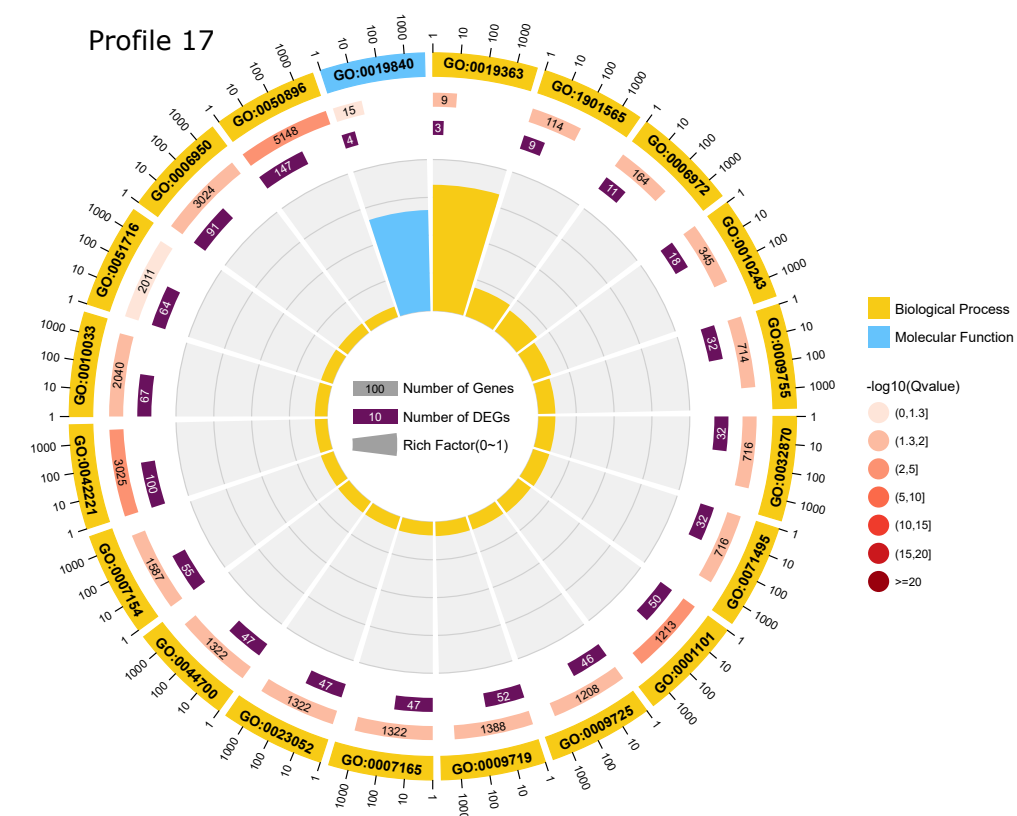

B

Profile 19

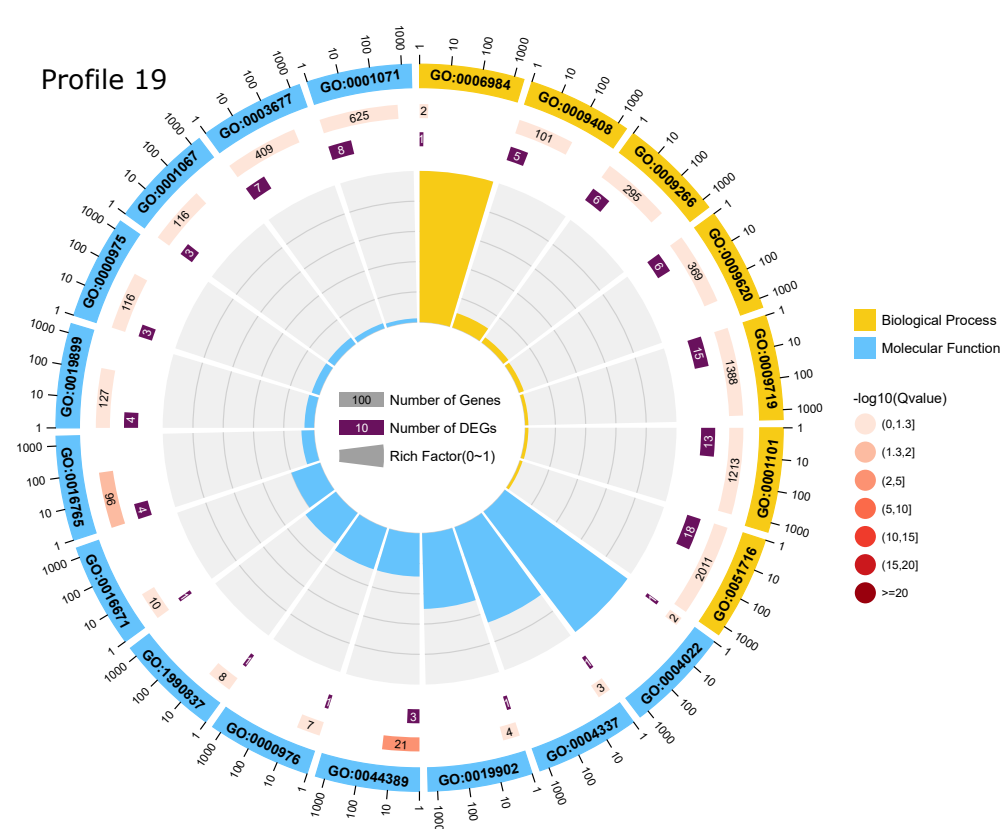

C

Profile 12

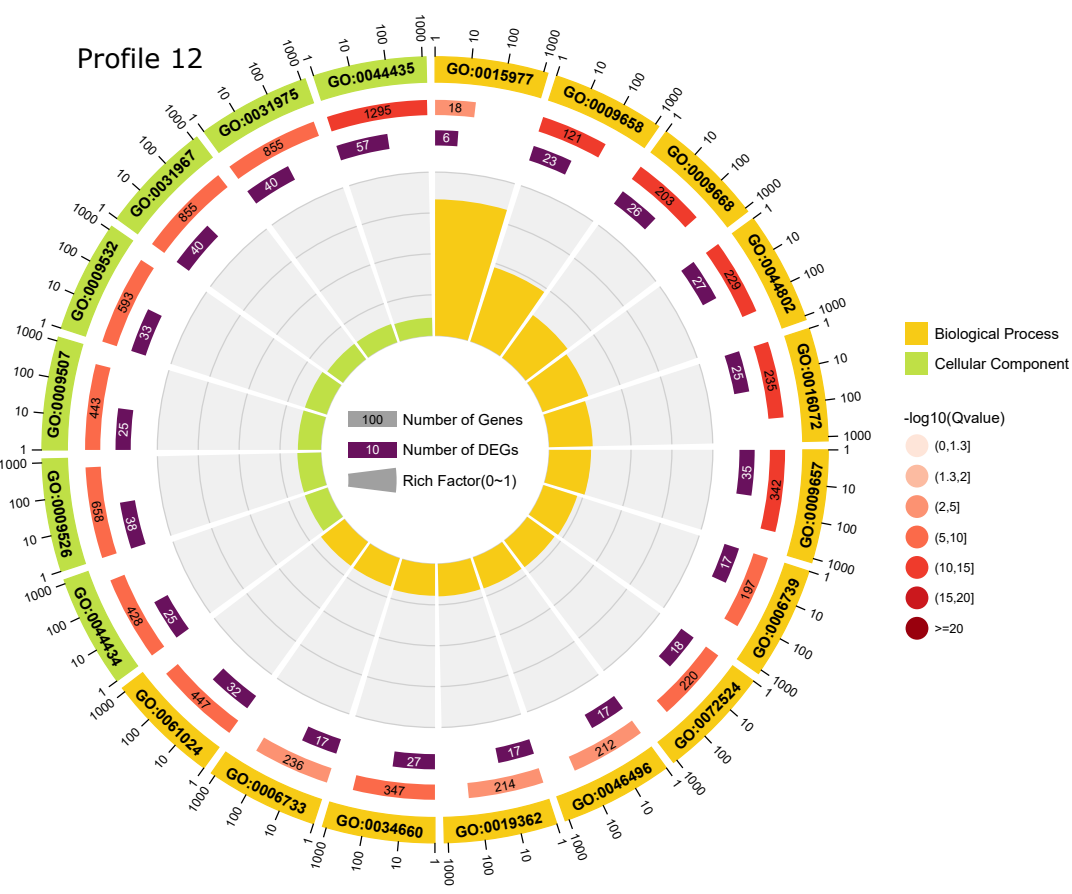

D

Profile 17

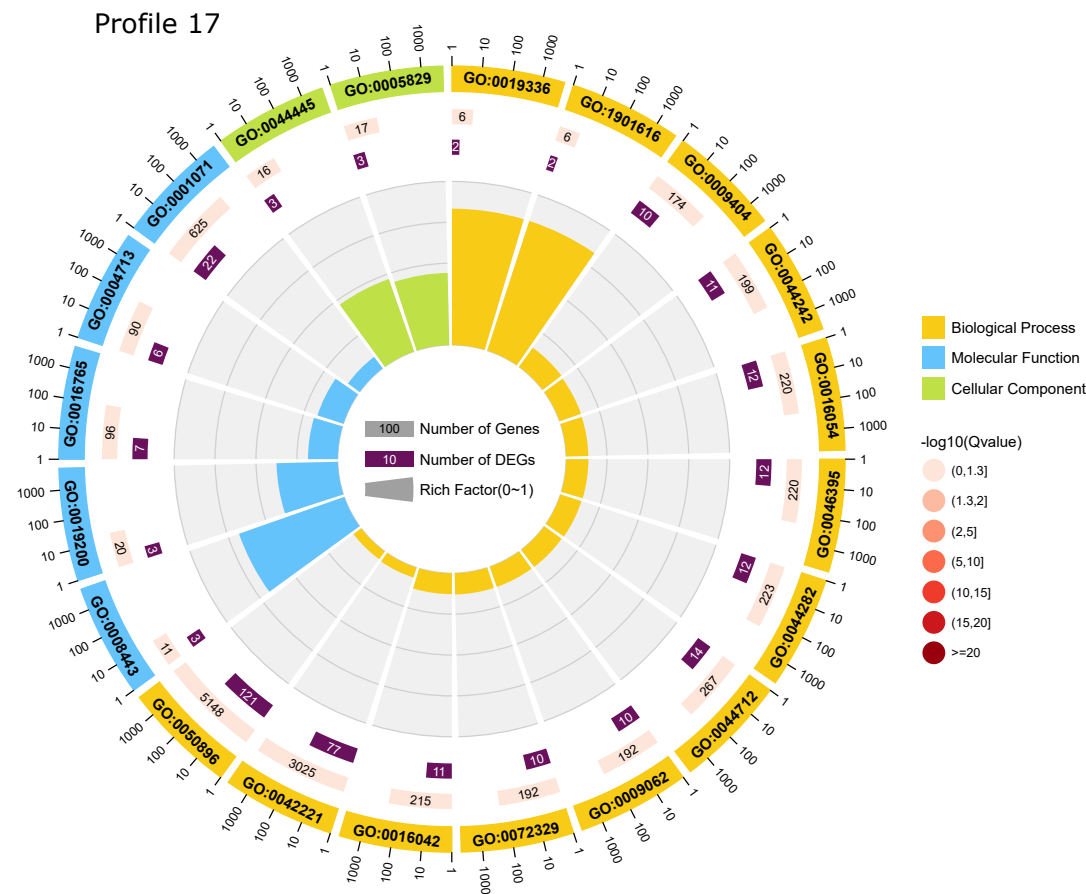

Supplement: Supplementary file 5 — Additional file 5. [file 12870_2022_3550_MOESM5_ESM.pdf]

A

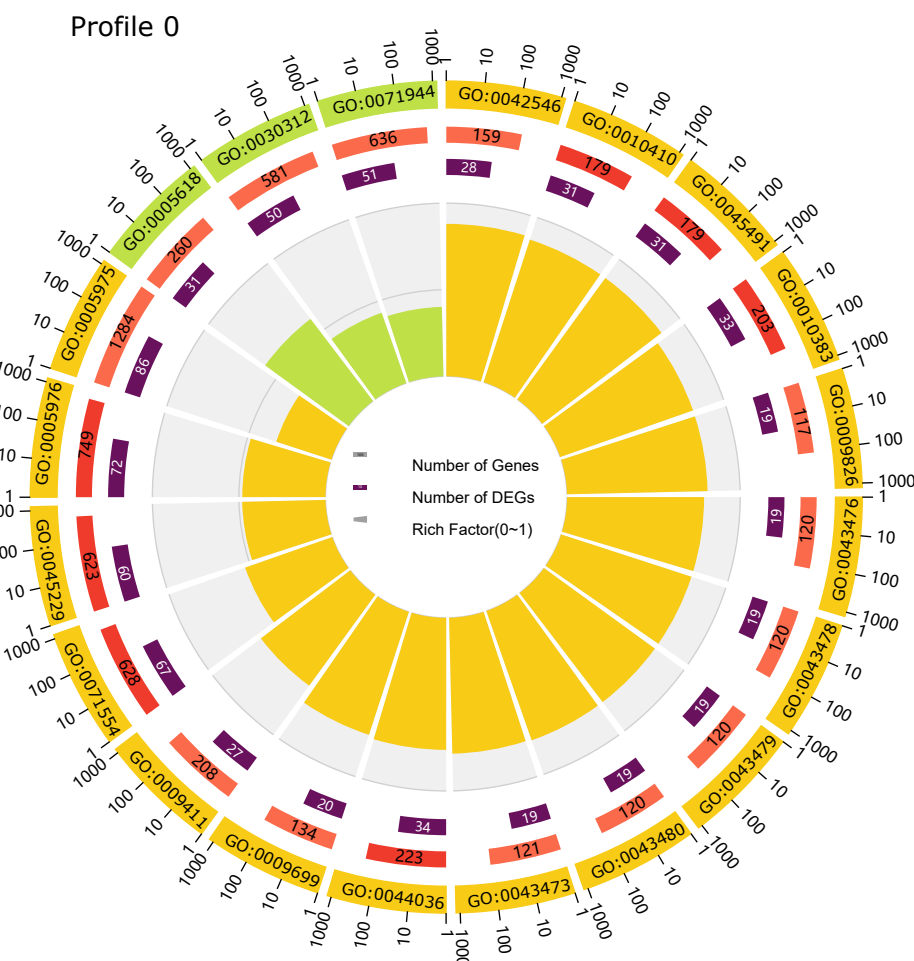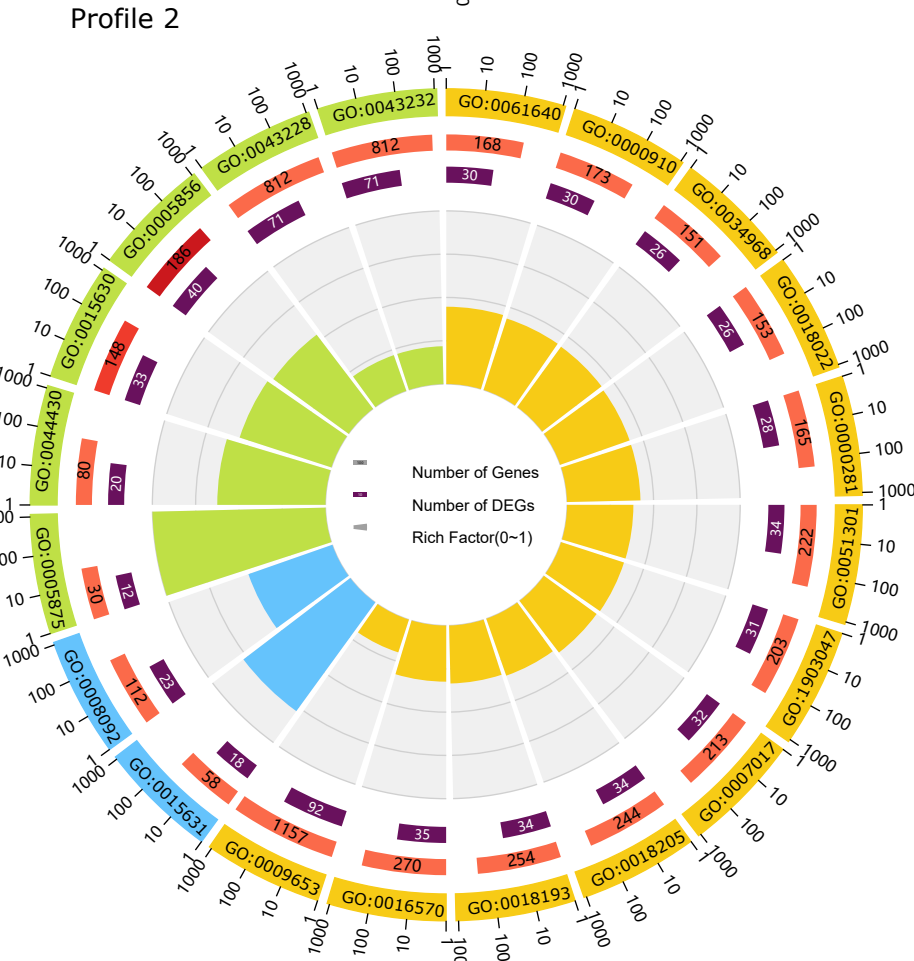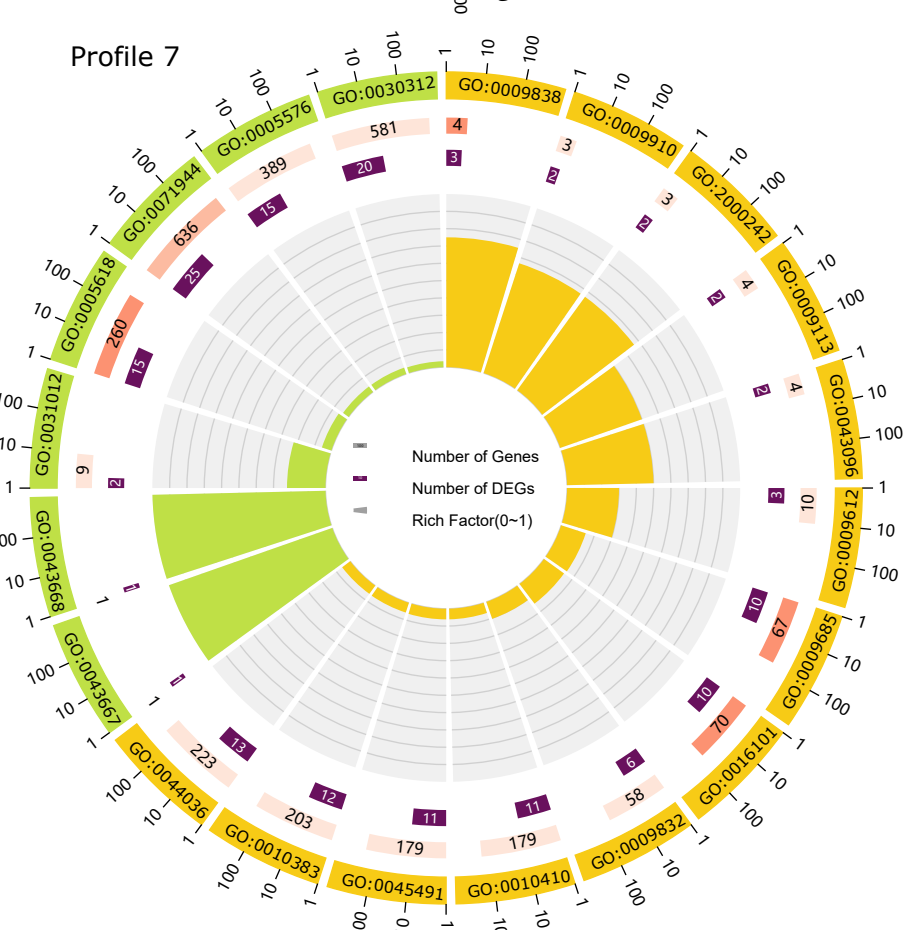

B

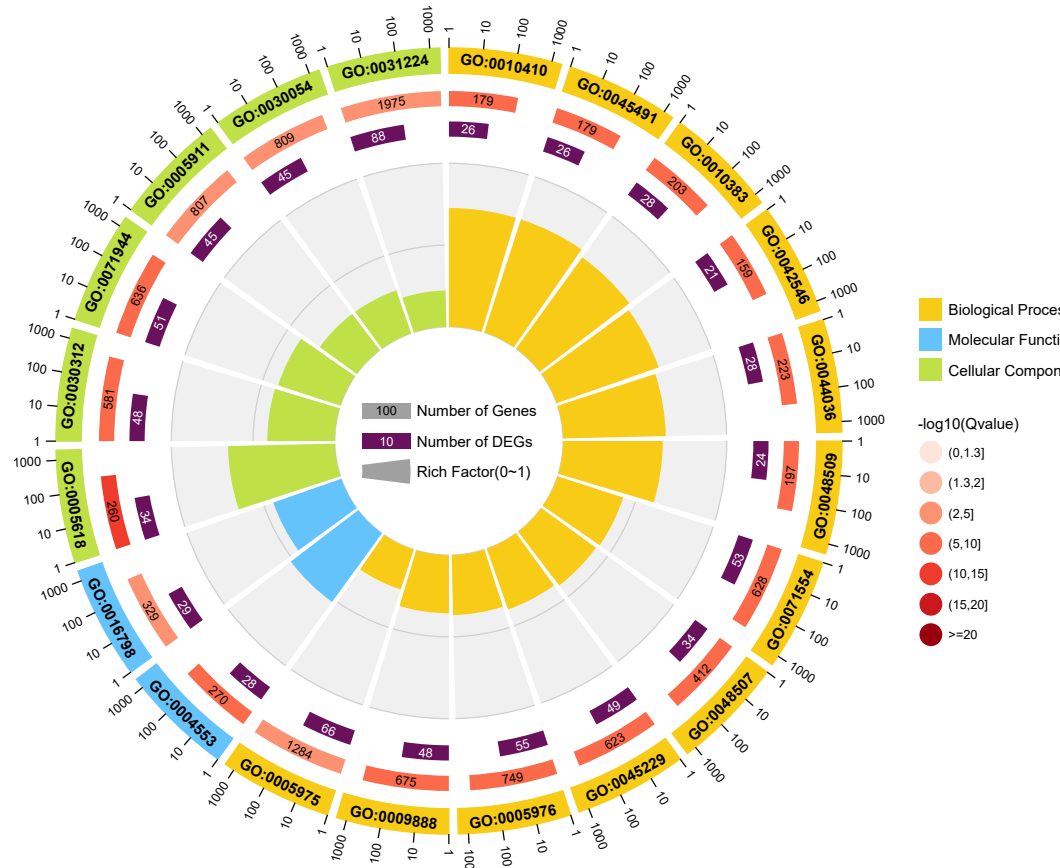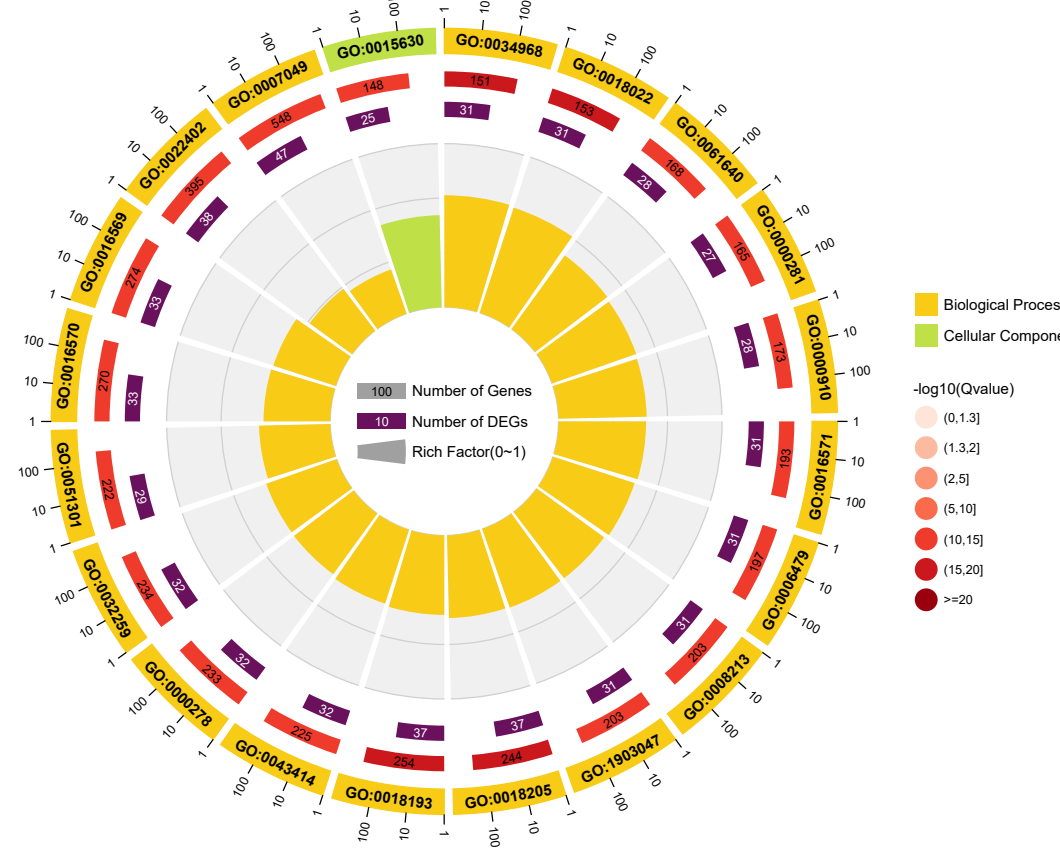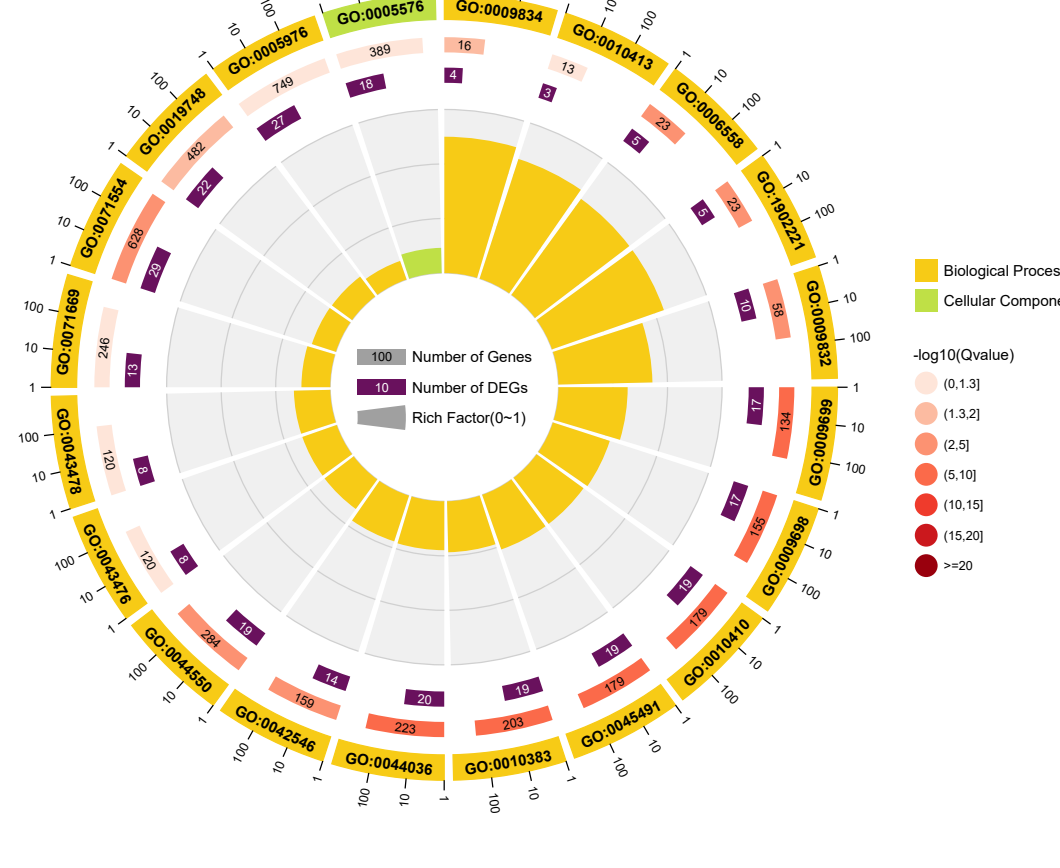

Supplement: Supplementary file 6 — Additional file 6. [file 12870_2022_3550_MOESM6_ESM.pdf]

A

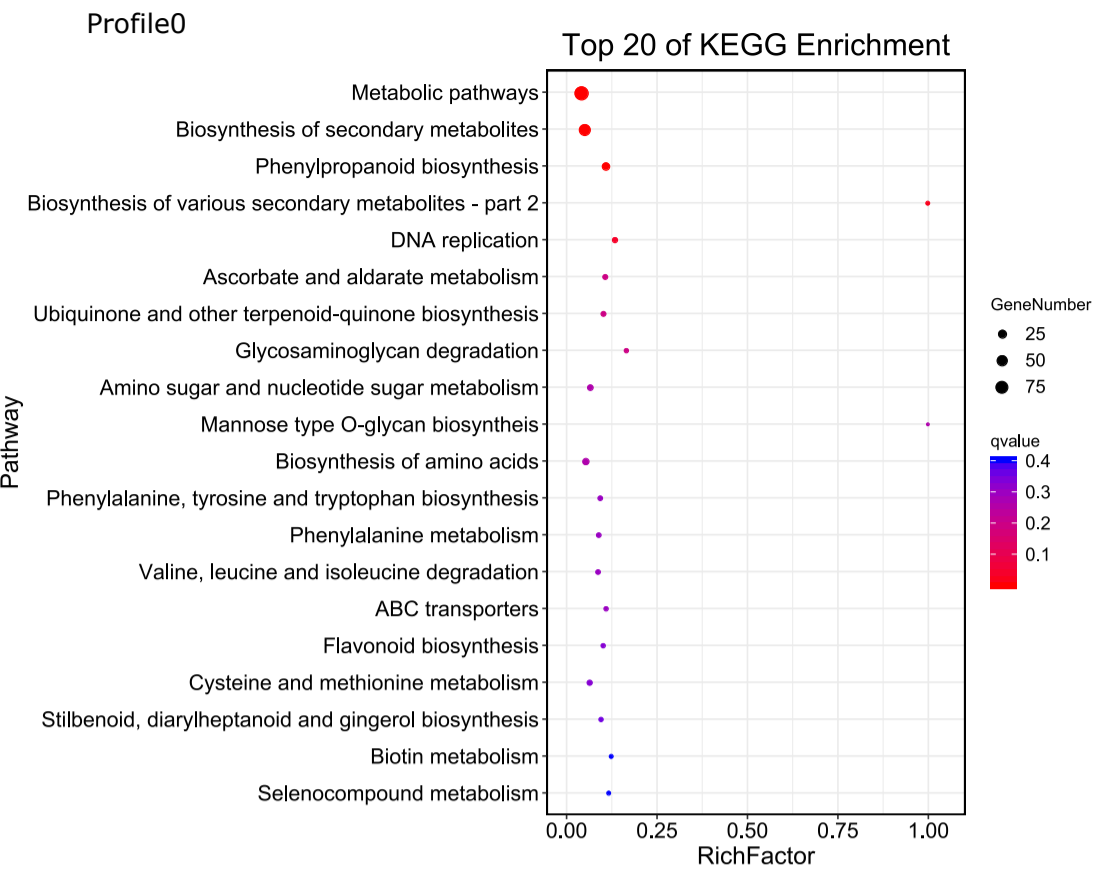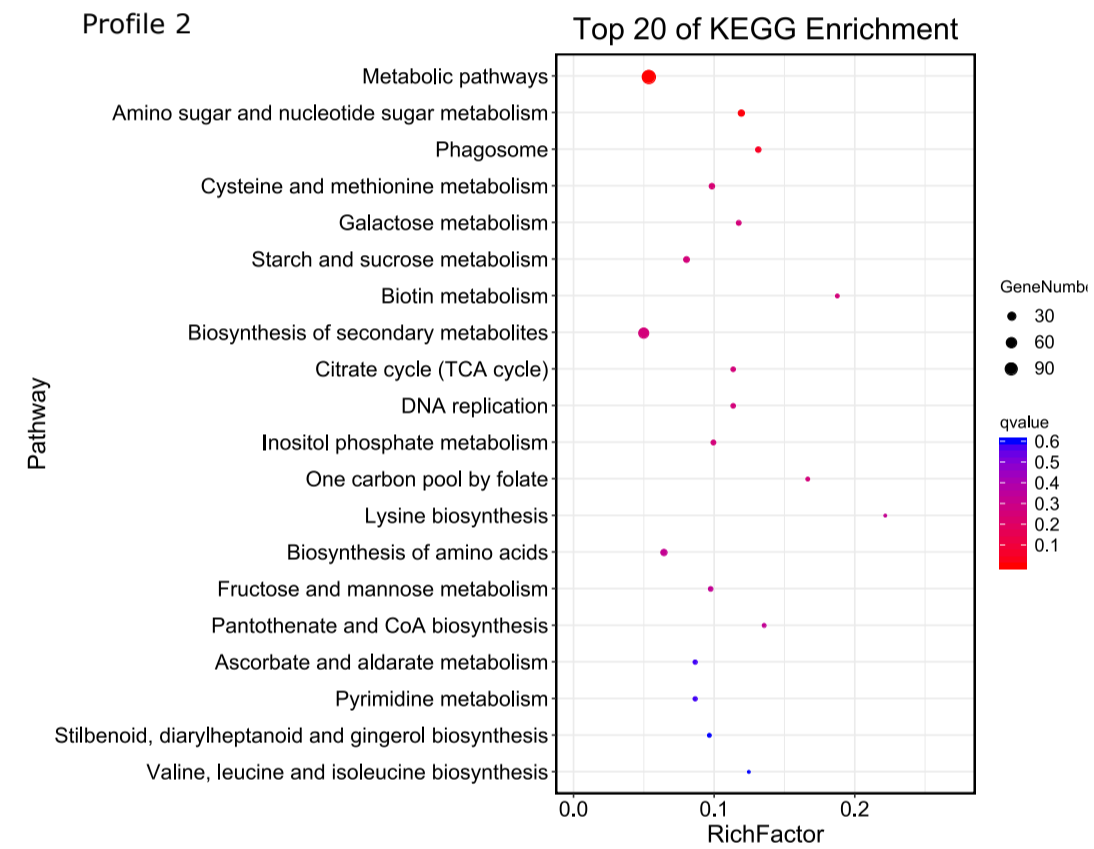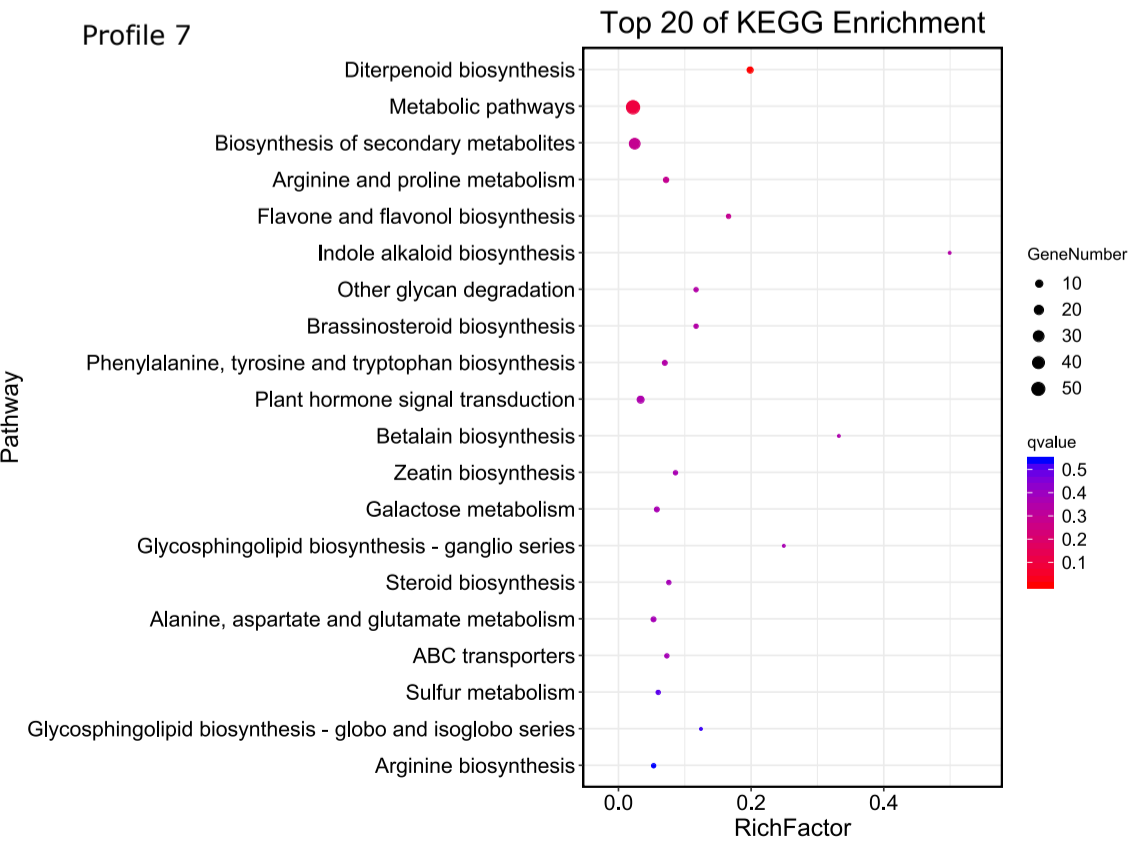

B

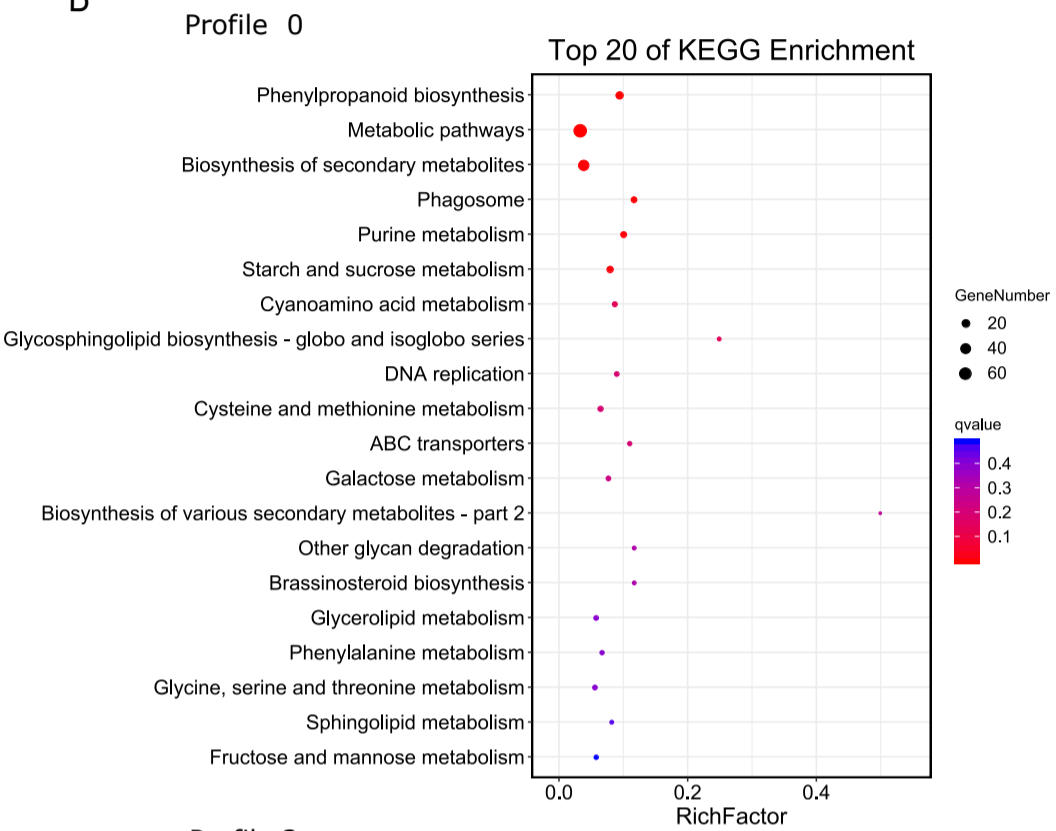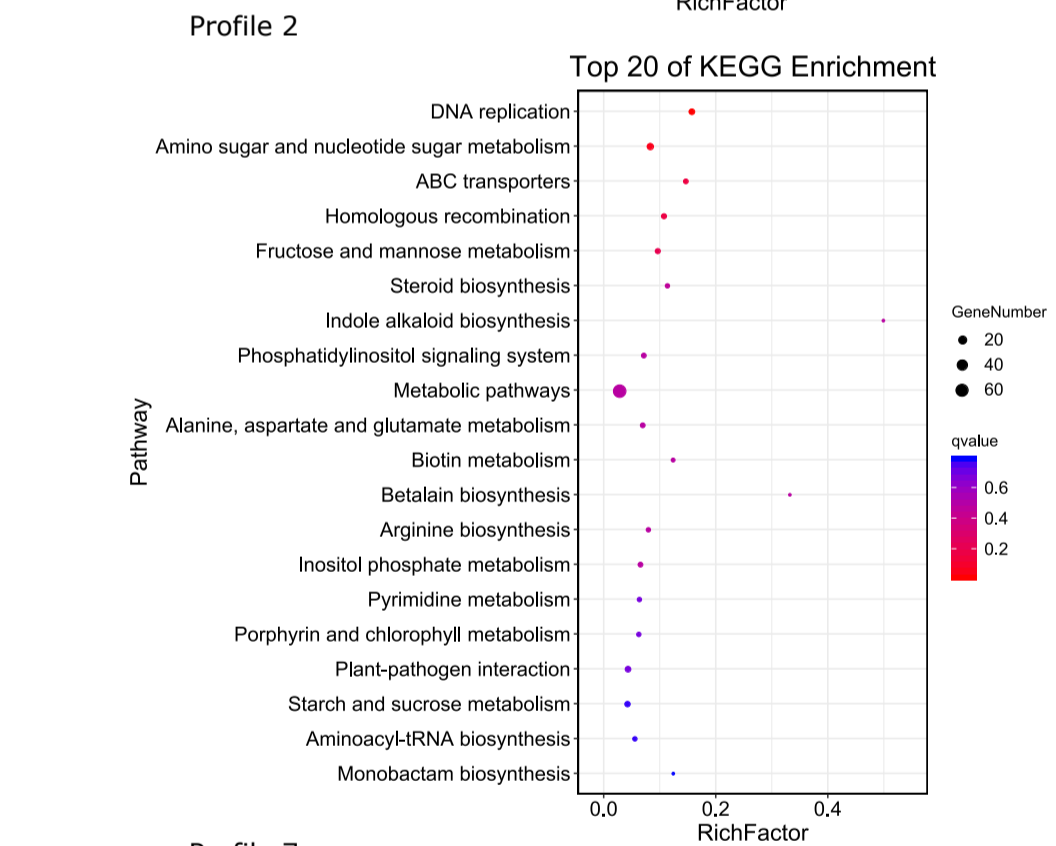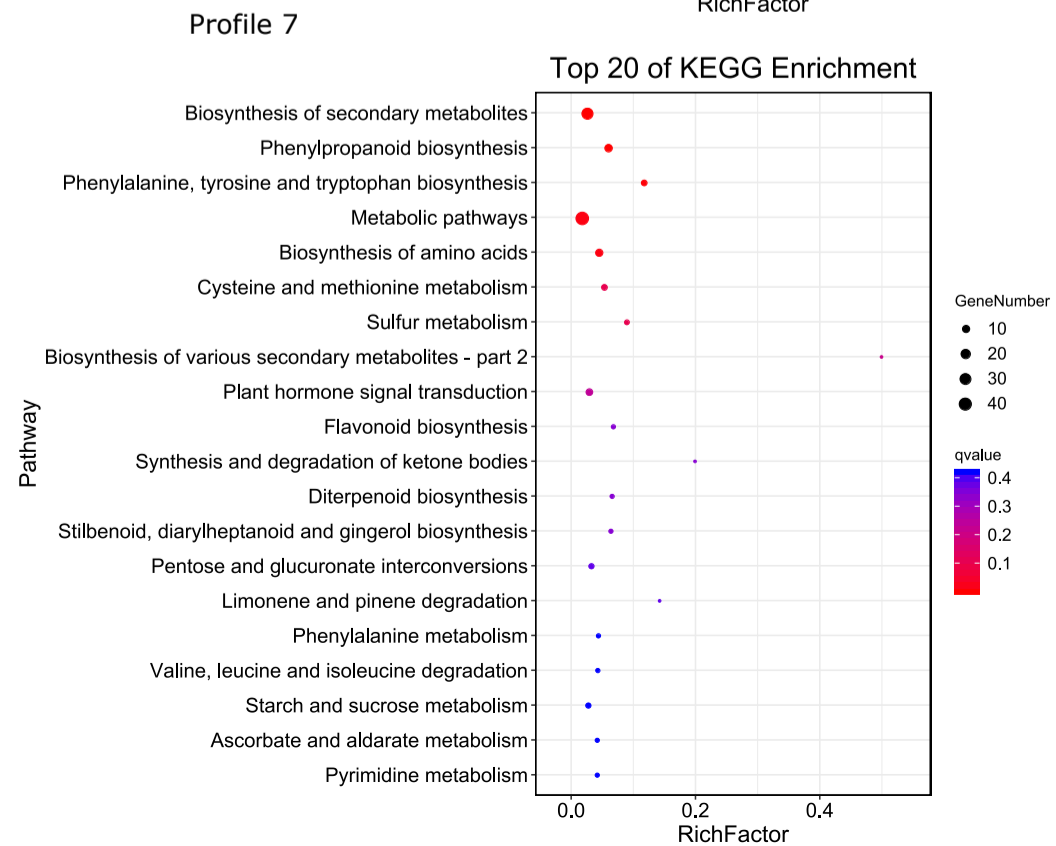

Supplement: Supplementary file 7 — Additional file 7. [file 12870_2022_3550_MOESM7_ESM.pdf]

A

Profile 12

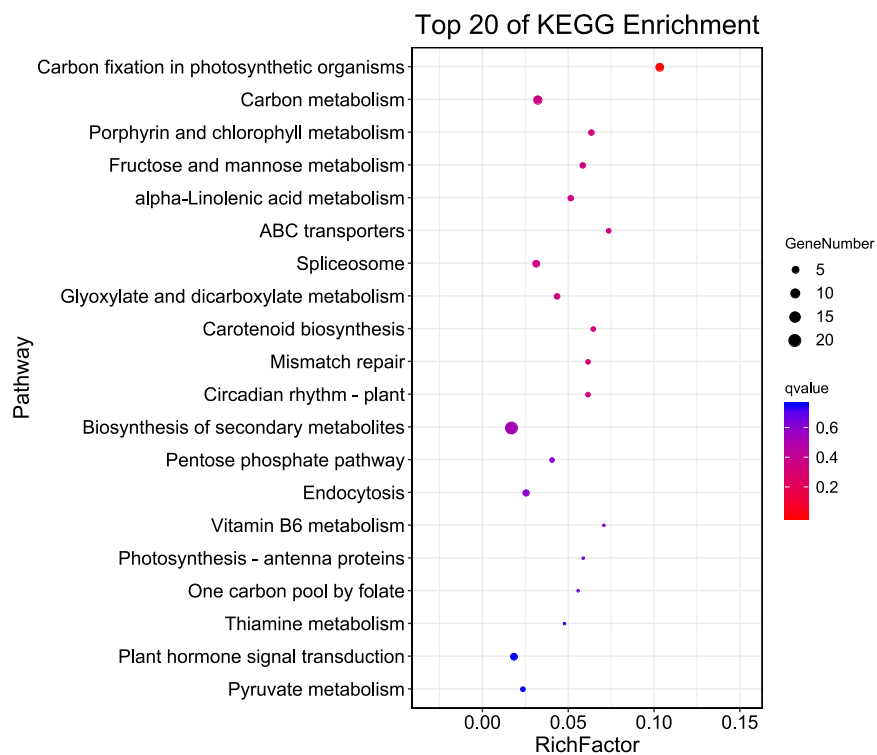

B

Profile 19

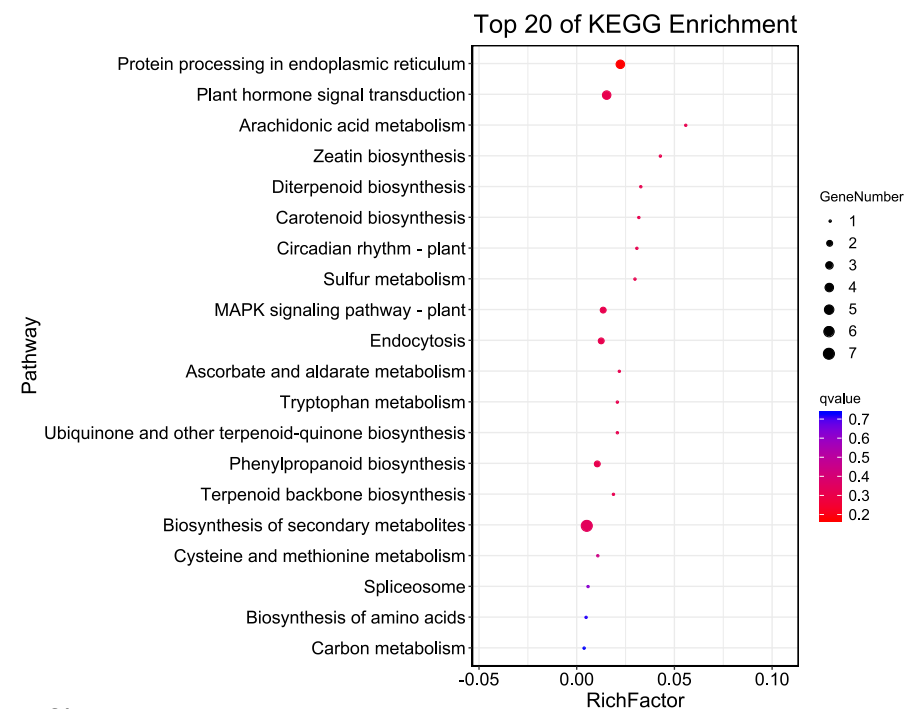

Profile 17

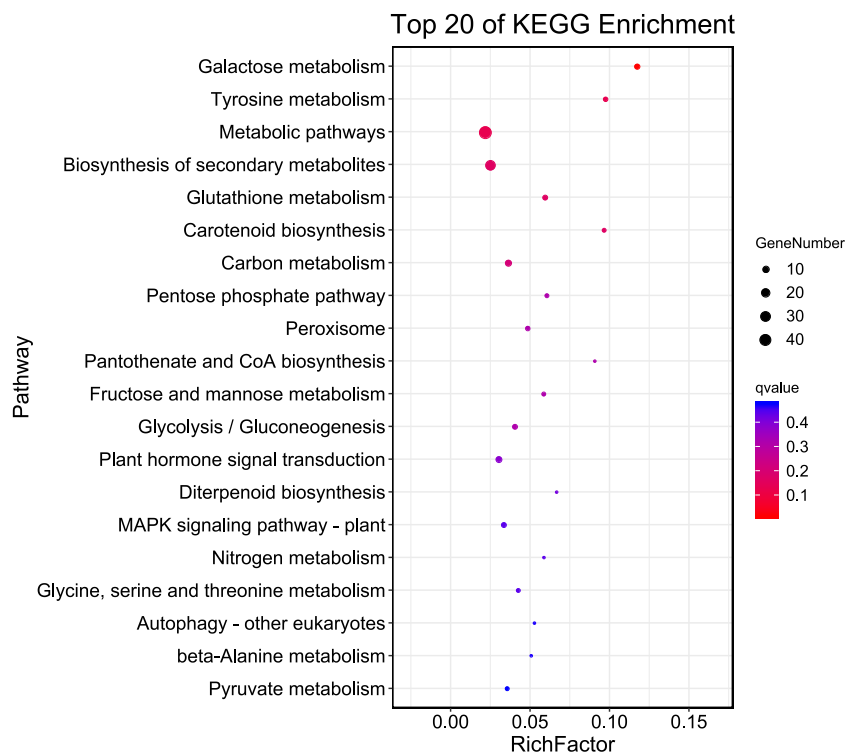

Profile 17

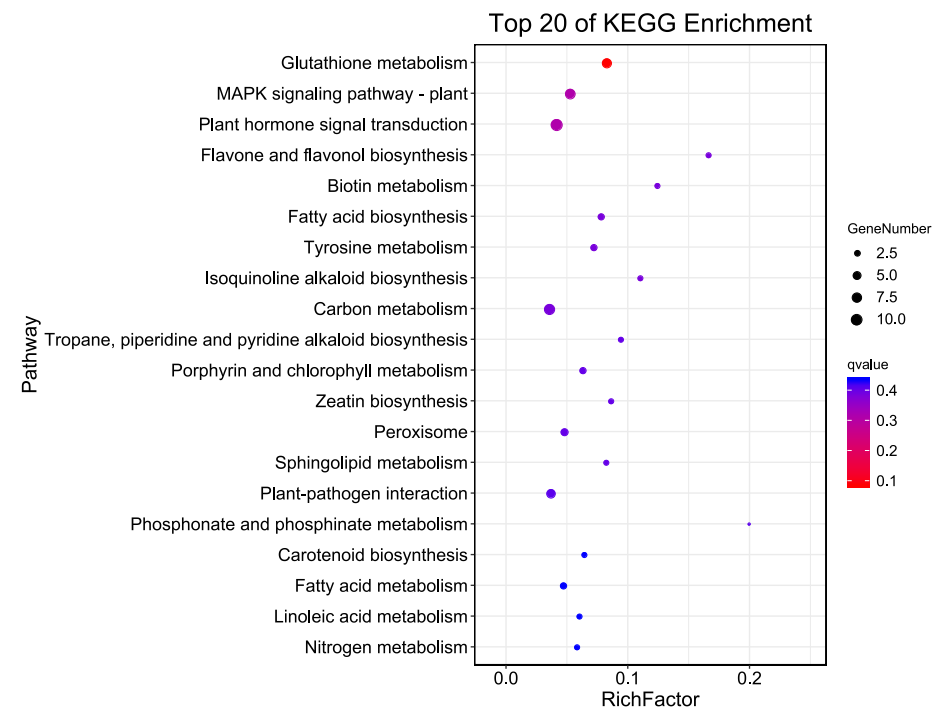

Supplement: Supplementary file 8 — Additional file 8. [file 12870_2022_3550_MOESM8_ESM.pdf]
