# Supplementary material for: Comparative transcriptomic analysis of two Cucumis melo var. saccharinus germplasms differing in fruit physical and chemical characteristics
Source: BMC Plant Biol. 2022 Apr 12;22:193. doi: 10.1186/s12870-022-03550-8 (PMC9004126; doi:10.1186/s12870-022-03550-8)
Supplement: Supplementary file 9 — Additional file 9. [file 12870_2022_3550_MOESM9_ESM.docx]

Table S1. Number of reads after filtering rRNA and low quality.

| Sample | Total | Unmapped(%) | Unique_Mapped(%) | Multiple_Mapped(%) | Total_Mapped(%) | Genes (%) |
| --- | --- | --- | --- | --- | --- | --- |
| Guimi10d-1 | 41409238 | 2960817 (7.15%) | 38016683 (91.81%) | 431738 (1.04%) | 38448421 (92.85%) | 18902 (85.78%) |
| Guimi10d-2 | 55866136 | 3799651 (6.80%) | 51460731 (92.11%) | 605754 (1.08%) | 52066485 (93.20%) | 15901 (72.16%) |
| Guimi10d-3 | 43065126 | 3060194 (7.11%) | 39554733 (91.85%) | 450199 (1.05%) | 40004932 (92.89%) | 18865 (85.61%) |
| Guimi20d-1 | 46690734 | 3178464 (6.81%) | 43018861 (92.14%) | 493409 (1.06%) | 43512270 (93.19%) | 18491 (83.92%) |
| Guimi20d-2 | 58166738 | 4722911 (8.12%) | 52766417 (90.72%) | 677410 (1.16%) | 53443827 (91.88%) | 18556 (84.21%) |
| Guimi20d-3 | 52048562 | 3093849 (5.94%) | 48354432 (92.90%) | 600281 (1.15%) | 48954713 (94.06%) | 18731 (85.01%) |
| Guimi30d-1 | 46338886 | 2626958 (5.67%) | 43172410 (93.17%) | 539518 (1.16%) | 43711928 (94.33%) | 19003 (86.24%) |
| Guimi30d-2 | 41996984 | 2939599 (7.00%) | 38573628 (91.85%) | 483757 (1.15%) | 39057385 (93.00%) | 18949 (86.00%) |
| Guimi30d-3 | 39166848 | 3867929 (9.88%) | 34898125 (89.10%) | 400794 (1.02%) | 35298919 (90.12%) | 18624 (84.52%) |
| Guimi40d-1 | 41206014 | 5494201 (13.33%) | 35210510 (85.45%) | 501303 (1.22%) | 35711813 (86.67%) | 17268 (78.37%) |
| Guimi40d-2 | 40237088 | 4811174 (11.96%) | 34997372 (86.98%) | 428542 (1.07%) | 35425914 (88.04%) | 18197 (82.58%) |
| Guimi40d-3 | 51570260 | 3032217 (5.88%) | 47913664 (92.91%) | 624379 (1.21%) | 48538043 (94.12%) | 18467 (83.81%) |
| Yaolong10d-1 | 41811208 | 2329698 (5.57%) | 39035812 (93.36%) | 445698 (1.07%) | 39481510 (94.43%) | 18888 (85.72%) |
| Yaolong10d-2 | 46309128 | 2745628 (5.93%) | 43064117 (92.99%) | 499383 (1.08%) | 43563500 (94.07%) | 18827 (85.44%) |
| Yaolong10d-3 | 57510440 | 3256888 (5.66%) | 53550004 (93.11%) | 703548 (1.22%) | 54253552 (94.34%) | 18270 (82.91%) |
| Yaolong20d-1 | 41854598 | 2476352 (5.92%) | 38918457 (92.98%) | 459789 (1.10%) | 39378246 (94.08%) | 18559 (84.23%) |
| Yaolong20d-2 | 47347664 | 3867794 (8.17%) | 42958654 (90.73%) | 521216 (1.10%) | 43479870 (91.83%) | 18724 (84.97%) |
| Yaolong20d-3 | 43549378 | 2831702 (6.50%) | 40220384 (92.36%) | 497292 (1.14%) | 40717676 (93.50%) | 18934 (85.93%) |
| Yaolong30d-1 | 54431504 | 3154690 (5.80%) | 50631281 (93.02%) | 645533 (1.19%) | 51276814 (94.20%) | 18594 (84.38%) |
| Yaolong30d-2 | 44752968 | 2752769 (6.15%) | 41481473 (92.69%) | 518726 (1.16%) | 42000199 (93.85%) | 17898 (81.23%) |
| Yaolong30d-3 | 41929212 | 3259651 (7.77%) | 38225188 (91.17%) | 444373 (1.06%) | 38669561 (92.23%) | 18573 (84.29%) |
| Yaolong40d-1 | 49162052 | 4105484 (8.35%) | 44464174 (90.44%) | 592394 (1.20%) | 45056568 (91.65%) | 18748 (85.08%) |
| Yaolong40d-2 | 43325662 | 2693007 (6.22%) | 40127423 (92.62%) | 505232 (1.17%) | 40632655 (93.78%) | 17769 (80.64%) |
| Yaolong40d-3 | 44230840 | 11305026 (25.56%) | 32538706 (73.57%) | 387108 (0.88%) | 32925814 (74.44%) | 18422 (83.60%) |

Table S2 Comparing the numble of DEGs of Guimi and Yaolong.

|  | UP | DOWN |
| --- | --- | --- |
| Guimi20d-vs-Guimi10d | 1141 | 1809 |
| Guimi30d-vs-Guimi20d | 1114 | 1377 |
| Guimi40d-vs-Guimi30d | 25 | 70 |
| Yaolong20d-vs-Yaolong10d | 1026 | 943 |
| Yaolong30d-vs-Yaolong20d | 344 | 1025 |
| Yaolong40d-vs-Yaolong30d | 69 | 154 |
| Guimi10d-vs-Yaolong10d | 153 | 64 |
| Guimi20d-vs-Yaolong20d | 448 | 839 |
| Guimi30d-vs-Yaolong30d | 445 | 410 |
| Guimi40d-vs-Yaolong40d | 221 | 237 |

Table S3. Top 30 Differentially expressed genes in Yaolong vs. Guimi at 10 DAP based on the FDR.

| id | guimi10d-1_fpkm | guimi10d-2_fpkm | guimi10d-3_fpkm | yaolong10d-1_fpkm | yaolong10d-2_fpkm | yaolong10d-3_fpkm | log2(fc) | PValue | FDR | Symbol |
| --- | --- | --- | --- | --- | --- | --- | --- | --- | --- | --- |
| MSTRG.24119 | 14.32 | 11.41 | 10.03 | 0.11 | 0.21 | 0.14 | -6.28057 | 8.22E-40 | 1.57E-35 | - |
| MSTRG.25411 | 13.43 | 11.24 | 6.44 | 0.33 | 0.42 | 0.46 | -4.6843 | 4.91E-24 | 4.70E-20 | - |
| ncbi_107991153 | 44.4 | 15.62 | 35.3 | 0 | 0 | 0 | -14.9555 | 2.82E-23 | 1.80E-19 | -- |
| MSTRG.13229 | 35.12 | 38.32 | 20.43 | 0 | 0 | 0 | -14.9334 | 3.82E-23 | 1.83E-19 | - |
| MSTRG.16877 | 46.82 | 44.15 | 22.28 | 0 | 0 | 0 | -15.2042 | 2.92E-21 | 1.12E-17 | - |
| MSTRG.1357 | 30.36 | 14.19 | 17.01 | 0 | 0 | 0 | -14.3247 | 3.71E-20 | 1.18E-16 | - |
| MSTRG.25362 | 24.42 | 17.67 | 14.55 | 0 | 0 | 0 | -14.2046 | 1.77E-18 | 4.85E-15 | - |
| ncbi_103498981 | 245.81 | 69.46 | 55.5 | 1.14 | 2.65 | 1.23 | -6.20669 | 3.86E-18 | 9.23E-15 | -- |
| MSTRG.24493 | 28.69 | 32.07 | 20.08 | 0 | 0 | 0 | -14.7178 | 7.21E-18 | 1.53E-14 | - |
| ncbi_103492870 | 43.47 | 113.97 | 209.68 | 0.26 | 0.48 | 0 | -8.95451 | 1.41E-17 | 2.71E-14 | H6H |
| MSTRG.21480 | 19.42 | 47.21 | 13.05 | 0 | 0 | 0 | -14.697 | 2.59E-15 | 4.51E-12 | - |
| MSTRG.22117 | 5.48 | 4.04 | 2.13 | 0 | 0.02 | 0.03 | -7.86419 | 1.71E-14 | 2.73E-11 | -- |
| ncbi_103495098 | 3.78 | 4.3 | 3.29 | 0 | 0.05 | 0.04 | -6.98109 | 2.36E-14 | 3.48E-11 | SULTR3;3 |
| MSTRG.20133 | 12.43 | 5.31 | 5.53 | 0 | 0 | 0 | -12.9212 | 2.90E-14 | 3.74E-11 | - |
| ncbi_103485478 | 10.2 | 13.84 | 6.67 | 0 | 0 | 0 | -13.3215 | 2.93E-14 | 3.74E-11 | PER51 |
| ncbi_103488897 | 4.79 | 5.84 | 3.32 | 62.96 | 126.5 | 263.89 | 5.022288 | 3.33E-14 | 3.99E-11 | At1g67980 |
| MSTRG.21459 | 0 | 0 | 0 | 13.68 | 12.26 | 14.57 | 13.72103 | 5.08E-14 | 5.72E-11 | -- |
| ncbi_103498736 | 12.27 | 22.45 | 7.3 | 0 | 0 | 0 | -13.7738 | 7.43E-14 | 7.90E-11 | BPA1 |
| MSTRG.16848 | 6.48 | 5.33 | 4.3 | 0 | 0 | 0 | -12.3907 | 1.60E-13 | 1.62E-10 | - |
| MSTRG.7848 | 0 | 0 | 0 | 10.19 | 6.98 | 16.37 | 13.44863 | 4.08E-13 | 3.90E-10 | NRPB10 |
| ncbi_103482719 | 6.83 | 7.96 | 16.52 | 0 | 0 | 0 | -13.3494 | 1.06E-12 | 9.69E-10 | TLP |
| MSTRG.16650 | 0 | 0 | 0 | 9.18 | 5.66 | 16.92 | 13.36996 | 1.30E-12 | 1.13E-09 | - |
| MSTRG.10414 | 21.21 | 16.43 | 8.88 | 1.2 | 0.67 | 1.27 | -3.88901 | 1.39E-12 | 1.16E-09 | - |
| MSTRG.22437 | 10.55 | 2.45 | 9.01 | 0 | 0 | 0 | -12.8409 | 2.32E-12 | 1.85E-09 | - |
| MSTRG.24278 | 8.42 | 17.21 | 6.68 | 0 | 0 | 0 | -13.3947 | 2.43E-12 | 1.86E-09 | - |
| ncbi_103490556 | 0 | 0 | 0 | 12.72 | 11.32 | 20.77 | 13.86657 | 6.91E-12 | 5.09E-09 | -- |
| ncbi_103487394 | 14.62 | 13.91 | 9.81 | 0 | 0.03 | 0.28 | -6.95044 | 9.19E-12 | 6.52E-09 | FMO1 |
| ncbi_103497259 | 9.71 | 4.17 | 4.32 | 0 | 0 | 0 | -12.5667 | 1.27E-11 | 8.67E-09 | CBSDUF1 |
| MSTRG.23748 | 0 | 0 | 0 | 3.77 | 8.28 | 8.97 | 12.77451 | 1.70E-11 | 1.12E-08 | - |
| ncbi_103486082 | 9.62 | 5.3 | 6.26 | 0 | 0 | 0.07 | -8.24113 | 2.97E-11 | 1.90E-08 | BIO3-BIO1 |

Table S4. Top 30 Differentially expressed genes in Yaolong vs. Guimi at 20 DAP based on the FDR.

| id | guimi20d-1_fpkm | guimi20d-2_fpkm | guimi20d-3_fpkm | yaolong20d-1_fpkm | yaolong20d-2_fpkm | yaolong20d-3_fpkm | log2(fc) | PValue | FDR | Symbol |
| --- | --- | --- | --- | --- | --- | --- | --- | --- | --- | --- |
| ncbi_103495267 | 261.45 | 221.39 | 216.83 | 4.32 | 4.55 | 3.8 | -5.78719 | 1.61E-89 | 3.08E-85 | HSP22.0 |
| ncbi_103491131 | 106.95 | 99.33 | 180.81 | 0.03 | 0.18 | 0.56 | -8.97359 | 2.82E-46 | 2.69E-42 | VIT_19s0014g04930 |
| ncbi_103488897 | 5.73 | 4.66 | 3.91 | 151.68 | 420.08 | 701.42 | 6.476277 | 5.62E-45 | 3.57E-41 | At1g67980 |
| MSTRG.25411 | 12.18 | 12.3 | 8.72 | 0.36 | 0.59 | 0.35 | -4.6746 | 5.90E-42 | 2.81E-38 | - |
| MSTRG.24119 | 17.41 | 16.72 | 10.84 | 0.07 | 0.1 | 0.35 | -6.43431 | 1.04E-37 | 3.98E-34 | - |
| ncbi_103492870 | 115.71 | 56.73 | 86.51 | 0.66 | 1.24 | 0.21 | -6.93929 | 7.23E-33 | 2.30E-29 | H6H |
| ncbi_103489180 | 3.92 | 3.56 | 3.21 | 18.78 | 23.66 | 26.43 | 2.687614 | 1.28E-31 | 3.49E-28 | TPT |
| MSTRG.8195 | 153.77 | 81.5 | 91.51 | 4.83 | 3.3 | 3.67 | -4.79146 | 2.28E-30 | 5.43E-27 | - |
| MSTRG.16877 | 72.01 | 57.34 | 53.26 | 0 | 0 | 0 | -15.8934 | 7.62E-26 | 1.61E-22 | - |
| ncbi_103504002 | 22.8 | 25 | 26.1 | 0 | 0 | 0.13 | -9.15092 | 8.99E-24 | 1.71E-20 | CYP88D6 |
| MSTRG.1357 | 29.98 | 36.39 | 20.74 | 0 | 0 | 0 | -14.8256 | 1.05E-23 | 1.82E-20 | - |
| ncbi_103483028 | 596.37 | 514.72 | 297.98 | 39.43 | 26.28 | 47.18 | -3.64175 | 2.06E-23 | 3.28E-20 | PDF2.3 |
| MSTRG.13229 | 33.36 | 22.05 | 18.5 | 0 | 0 | 0 | -14.5885 | 6.01E-23 | 8.82E-20 | - |
| ncbi_107991153 | 20.84 | 36.61 | 38.02 | 0 | 0 | 0 | -14.9578 | 1.07E-22 | 1.46E-19 | -- |
| ncbi_103498616 | 504.2 | 1266.24 | 556.63 | 20.84 | 35.25 | 62.95 | -4.289 | 4.51E-22 | 5.51E-19 | HSP22.0 |
| ncbi_103497680 | 979.16 | 1050.96 | 1370.67 | 43.39 | 86.72 | 134.27 | -3.68518 | 4.62E-22 | 5.51E-19 | BAG6 |
| MSTRG.25362 | 31.99 | 35.59 | 23.09 | 0 | 0 | 0 | -14.8834 | 1.26E-21 | 1.41E-18 | - |
| MSTRG.24493 | 40.83 | 48.95 | 33.55 | 0 | 0 | 0 | -15.3272 | 3.59E-21 | 3.81E-18 | - |
| MSTRG.5874 | 17.15 | 17.37 | 15.44 | 2.17 | 3.59 | 4.65 | -2.2628 | 7.27E-21 | 7.30E-18 | - |
| MSTRG.21480 | 36.96 | 30.09 | 20.92 | 0 | 0 | 0 | -14.8398 | 1.26E-19 | 1.21E-16 | - |
| MSTRG.16650 | 0 | 0 | 0 | 5.91 | 11.52 | 16.72 | 13.47464 | 5.91E-19 | 5.37E-16 | - |
| ncbi_103493476 | 1.59 | 2.36 | 1.65 | 23.12 | 49.69 | 17.9 | 4.017763 | 3.37E-18 | 2.92E-15 | DTX19 |
| ncbi_103488214 | 16.81 | 13.77 | 10.94 | 60.03 | 71.47 | 64.7 | 2.240447 | 4.98E-18 | 4.13E-15 | AIG2LD |
| ncbi_103488319 | 19.5 | 33.77 | 36.4 | 210.5 | 340.2 | 591.02 | 3.67044 | 9.96E-18 | 7.91E-15 | AUX28 |
| MSTRG.24278 | 22.03 | 23.21 | 15.25 | 0 | 0 | 0 | -14.2994 | 1.46E-17 | 1.12E-14 | - |
| ncbi_103491327 | 0 | 0 | 0 | 43.29 | 89.77 | 11.55 | 15.55685 | 3.02E-17 | 2.21E-14 | At5g64080 |
| ncbi_107991740 | 15.4 | 26.49 | 9.98 | 116.83 | 435.93 | 642.45 | 4.52622 | 3.68E-17 | 2.60E-14 | -- |
| ncbi_103500475 | 21.5 | 26.23 | 34.76 | 2.34 | 3.91 | 3.94 | -3.01707 | 6.22E-17 | 4.23E-14 | CYP71AP13 |
| MSTRG.17622 | 14.09 | 15.03 | 11.78 | 0 | 0 | 0 | -13.7349 | 8.47E-17 | 5.57E-14 | PMAT1 |
| ncbi_103485397 | 0.03 | 0.03 | 0 | 8.79 | 8.76 | 11.83 | 8.935656 | 9.38E-17 | 5.96E-14 | -- |

Table S5. Top 30 Differentially expressed genes in Yaolong vs. Guimi at 30 DAP based on the FDR.

| id | guimi30d-1_fpkm | guimi30d-2_fpkm | guimi30d-3_fpkm | yaolong30d-1_fpkm | yaolong30d-2_fpkm | yaolong30d-3_fpkm | log2(fc) | PValue | FDR | Symbol |
| --- | --- | --- | --- | --- | --- | --- | --- | --- | --- | --- |
| ncbi_103488897 | 3.38 | 3.64 | 4.28 | 305.1 | 339.13 | 269.41 | 6.337231 | ###### | ###### | At1g67980 |
| ncbi_103484107 | 237.83 | 322.23 | 269.68 | 18.02 | 12.65 | 9.42 | -4.37135 | 1.21E-41 | 1.13E-37 | -- |
| ncbi_103487187 | 20.23 | 24.84 | 21.44 | 1.08 | 1.43 | 0.92 | -4.27729 | 5.90E-39 | 3.66E-35 | CYP716A15 |
| ncbi_103488439 | 8.62 | 8.52 | 9.72 | 290.45 | 237.56 | 123.83 | 4.600987 | 2.17E-38 | 1.01E-34 | CRSP |
| MSTRG.24119 | 24.17 | 12.82 | 23.96 | 0.49 | 0.36 | 0.14 | -5.94405 | 1.08E-32 | 4.00E-29 | - |
| ncbi_103485397 | 0.06 | 0.03 | 0.12 | 13.93 | 23.04 | 14.71 | 7.943073 | 1.83E-29 | 5.67E-26 | -- |
| MSTRG.25411 | 24.91 | 17.98 | 21.99 | 0.62 | 0.94 | 0.22 | -5.18782 | 1.78E-26 | 4.72E-23 | - |
| MSTRG.16877 | 60.92 | 59.01 | 59.36 | 0 | 0 | 0 | -15.867 | 2.60E-26 | 6.03E-23 | - |
| MSTRG.13229 | 48.7 | 30.05 | 46.14 | 0 | 0 | 0 | -15.3453 | 8.87E-26 | 1.83E-22 | - |
| ncbi_107991153 | 80.96 | 35.18 | 60.19 | 0 | 0 | 0 | -15.843 | 2.22E-25 | 4.13E-22 | -- |
| MSTRG.1357 | 28.63 | 25.69 | 34.63 | 0 | 0 | 0 | -14.8557 | 1.95E-24 | 3.29E-21 | - |
| MSTRG.24493 | 47.99 | 59.5 | 44.28 | 0 | 0 | 0 | -15.6266 | 2.92E-22 | 4.52E-19 | - |
| MSTRG.21480 | 38.22 | 43.77 | 35.67 | 0 | 0 | 0 | -15.2593 | 1.22E-21 | 1.74E-18 | - |
| ncbi_103495878 | 0.53 | 1.04 | 0.37 | 32.76 | 84.86 | 16.91 | 6.115727 | 9.58E-21 | 1.27E-17 | ZOX1 |
| MSTRG.7848 | 0.03 | 0.07 | 0 | 10.1 | 14.66 | 14.45 | 8.615078 | 1.12E-20 | 1.39E-17 | NRPB10 |
| ncbi_103485707 | 7.39 | 4.77 | 7.69 | 0.07 | 0.09 | 0.2 | -5.785 | 1.76E-20 | 2.04E-17 | YSL5 |
| MSTRG.10414 | 19.51 | 16.59 | 29.17 | 2.05 | 2.07 | 2.53 | -3.29499 | 1.04E-19 | 1.14E-16 | - |
| MSTRG.25362 | 17.07 | 27.6 | 22.76 | 0 | 0 | 0 | -14.4561 | 1.28E-19 | 1.32E-16 | - |
| MSTRG.5817 | 14.27 | 9.42 | 18.15 | 0 | 0 | 0 | -13.7676 | 7.52E-19 | 7.36E-16 | - |
| MSTRG.5668 | 6.7 | 7.57 | 6.96 | 0.16 | 0.26 | 0.16 | -5.19391 | 7.92E-19 | 7.36E-16 | - |
| ncbi_103500475 | 90.09 | 51.74 | 34.91 | 5.36 | 3.45 | 2.97 | -3.90722 | 9.44E-19 | 8.36E-16 | CYP71AP13 |
| MSTRG.20317 | 0.31 | 0.35 | 0.1 | 53.5 | 21.17 | 14.15 | 6.868741 | 1.27E-18 | 1.07E-15 | - |
| MSTRG.24737 | 15.46 | 9.25 | 12.98 | 0 | 0 | 0 | -13.6169 | 1.92E-18 | 1.55E-15 | - |
| ncbi_103491131 | 15.71 | 29.39 | 5.08 | 0.12 | 0.12 | 0 | -7.70793 | 2.09E-18 | 1.62E-15 | VIT_19s0014g04930 |
| ncbi_103490556 | 0 | 0 | 0 | 19.9 | 22.06 | 18.6 | 14.30112 | 4.00E-18 | 2.97E-15 | -- |
| MSTRG.20133 | 17.83 | 8.9 | 17.1 | 0 | 0 | 0 | -13.8347 | 4.80E-18 | 3.43E-15 | - |
| MSTRG.15844 | 12.16 | 10.07 | 11.61 | 0 | 0 | 0 | -13.4615 | 2.00E-17 | 1.38E-14 | - |
| ncbi_103492983 | 33.09 | 33.9 | 97.89 | 1275.41 | 976.15 | 704.36 | 4.164119 | 3.24E-17 | 2.15E-14 | GSTU25 |
| MSTRG.24278 | 17 | 23.71 | 15.83 | 0 | 0 | 0 | -14.202 | 3.41E-17 | 2.18E-14 | - |
| MSTRG.14038 | 12.79 | 7.67 | 7.23 | 0 | 0 | 0 | -13.1721 | 3.91E-17 | 2.42E-14 | - |

Table S6. Top 30 Differentially expressed genes in Yaolong vs. Guimi at 40 DAP based on the FDR.

| id | guimi40d-1_fpkm | guimi40d-2_fpkm | guimi40d-3_fpkm | yaolong40d-1_fpkm | yaolong40d-2_fpkm | yaolong40d-3_fpkm | log2(fc) | PValue | FDR | Symbol |
| --- | --- | --- | --- | --- | --- | --- | --- | --- | --- | --- |
| MSTRG.25411 | 18.62 | 20.4 | 25.88 | 0.89 | 0.57 | 1.03 | -4.704 | 8.15E-40 | 1.44E-35 | - |
| ncbi_103488897 | 0.97 | 4.56 | 2.44 | 146.99 | 201.7 | 176.33 | 6.041649 | 7.42E-35 | 6.57E-31 | At1g67980 |
| MSTRG.16877 | 38.05 | 51.51 | 53.11 | 0 | 0 | 0 | -15.5374 | 1.44E-24 | 8.07E-21 | - |
| MSTRG.13229 | 46.28 | 33.58 | 43.89 | 0 | 0 | 0 | -15.3321 | 1.82E-24 | 8.07E-21 | - |
| ncbi_103485397 | 0 | 0 | 0.11 | 16.7 | 17.9 | 22.98 | 9.03192 | 6.06E-23 | 2.14E-19 | -- |
| MSTRG.1357 | 47.74 | 28.14 | 31.43 | 0 | 0 | 0 | -15.1265 | 1.25E-22 | 3.67E-19 | - |
| ncbi_107991153 | 16.7 | 47.92 | 84.33 | 0 | 0 | 0 | -15.5995 | 3.88E-22 | 8.78E-19 | -- |
| ncbi_103486082 | 10.73 | 11.9 | 10.67 | 0.28 | 0.24 | 0.31 | -5.32627 | 3.97E-22 | 8.78E-19 | BIO3-BIO1 |
| MSTRG.7848 | 0 | 0 | 0.09 | 24.62 | 22.44 | 14.08 | 9.407976 | 9.81E-20 | 1.93E-16 | NRPB10 |
| MSTRG.21480 | 20.86 | 28.87 | 23.54 | 0 | 0 | 0 | -14.576 | 6.53E-19 | 1.10E-15 | - |
| MSTRG.9975 | 16.73 | 24.71 | 22.25 | 0 | 0 | 0 | -14.3738 | 6.82E-19 | 1.10E-15 | - |
| MSTRG.24493 | 42.84 | 41.5 | 46.09 | 0 | 0 | 0.1 | -10.3491 | 6.83E-18 | 1.01E-14 | - |
| MSTRG.23362 | 0 | 0 | 0 | 10.71 | 9.18 | 9.33 | 13.24971 | 9.10E-18 | 1.24E-14 | - |
| MSTRG.23748 | 0 | 0 | 0 | 10.2 | 14.06 | 19.65 | 13.8373 | 2.42E-17 | 3.06E-14 | - |
| ncbi_103495878 | 0 | 0.58 | 0.39 | 21.13 | 76.75 | 53.43 | 7.285307 | 2.69E-17 | 3.17E-14 | ZOX1 |
| MSTRG.17622 | 12.43 | 16.15 | 18.13 | 0 | 0 | 0 | -13.9265 | 3.57E-17 | 3.95E-14 | PMAT1 |
| MSTRG.21459 | 0 | 0 | 0 | 16.1 | 14.68 | 12.84 | 13.82774 | 9.15E-17 | 9.53E-14 | -- |
| MSTRG.16848 | 4.58 | 6.27 | 6.99 | 0.07 | 0.08 | 0 | -6.89401 | 2.08E-16 | 2.04E-13 | - |
| MSTRG.16423 | 0 | 0.08 | 0.08 | 16.92 | 20.09 | 5.47 | 8.052568 | 3.17E-16 | 2.96E-13 | - |
| ncbi_103490556 | 0 | 0 | 0 | 24.98 | 22.55 | 14.78 | 14.34221 | 3.35E-16 | 2.96E-13 | -- |
| ncbi_103492106 | 262.55 | 479.84 | 333.21 | 29.61 | 57.74 | 51.56 | -2.95292 | 3.91E-16 | 3.29E-13 | HSP70 |
| MSTRG.6454 | 17.43 | 15.42 | 13.42 | 0 | 0 | 0 | -13.9128 | 6.04E-16 | 4.86E-13 | - |
| MSTRG.17633 | 0 | 0 | 0 | 33.14 | 26.36 | 44.12 | 15.07598 | 7.44E-16 | 5.72E-13 | - |
| MSTRG.6269 | 8.74 | 12.6 | 13.65 | 1.66 | 1.22 | 1.73 | -2.9241 | 1.06E-15 | 7.83E-13 | - |
| MSTRG.25362 | 5.47 | 16.4 | 15.01 | 0 | 0 | 0 | -13.5856 | 1.15E-15 | 8.12E-13 | - |
| ncbi_103491847 | 1.85 | 1.51 | 1.55 | 0.1 | 0.03 | 0.04 | -4.85212 | 1.95E-15 | 1.32E-12 | -- |
| ncbi_103495273 | 1.02 | 1.96 | 1.53 | 16.63 | 15.5 | 10.58 | 3.243375 | 2.37E-15 | 1.55E-12 | UGT84A13 |
| ncbi_103500842 | 0 | 0 | 0 | 17.83 | 6.5 | 25.67 | 14.02468 | 5.34E-15 | 3.38E-12 | glnA |
| MSTRG.13951 | 2.61 | 5.5 | 5.66 | 0 | 0 | 0 | -12.1643 | 1.13E-14 | 6.88E-12 | - |
| MSTRG.24119 | 4.24 | 20.02 | 26.27 | 0.42 | 0.31 | 0.82 | -5.0268 | 1.52E-14 | 8.70E-12 | - |
